# Tetrahydroisoquinolines affect the whole-cell phenotype of *Mycobacterium tuberculosis* by inhibiting the ATP-dependent MurE ligase

**DOI:** 10.1093/jac/dkv010

**Published:** 2015-02-04

**Authors:** Juan D. Guzman, Thomas Pesnot, Diana A. Barrera, Heledd M. Davies, Eleanor McMahon, Dimitrios Evangelopoulos, Parisa N. Mortazavi, Tulika Munshi, Arundhati Maitra, Eleanor D. Lamming, Richard Angell, Markus C. Gershater, Joanna M. Redmond, Deborah Needham, John M. Ward, Luis E. Cuca, Helen C. Hailes, Sanjib Bhakta

**Affiliations:** 1 Mycobacteria Research Laboratory, Institute of Structural and Molecular Biology, Department of Biological Sciences, Birkbeck, University of London, Malet Street, London WC1E 7HX, UK; 2 Department of Chemistry, University College London, 20 Gordon Street, London WC1H 0AJ, UK; 3 Departamento de Química, Facultad de Ciencias, Universidad Nacional de Colombia, Carrera 30 No. 45-03, Bogotá, Colombia; 4 Drug Discovery Group, UCL School of Pharmacy, 29-39 Brunswick Square, London WC1N 1AX, UK; 5 The Advanced Centre for Biochemical Engineering, University College London, Gordon Street, London WC1H 0AH, UK; 6 Department of Medicinal Chemistry, GlaxoSmithKline Medicines Research Centre, Gunnels Wood Road, Stevenage, Hertfordshire SG1 2NY, UK

**Keywords:** tuberculosis, TB, *Mycobacterium tuberculosis*, drug resistance, cell wall peptidoglycan, aporphine alkaloids, SPOTi

## Abstract

**Objectives:**

(*S*)-Leucoxine, isolated from the Colombian Lauraceae tree *Rhodostemonodaphne crenaticupula* Madriñan, was found to inhibit the growth of *Mycobacterium tuberculosis* H_37_Rv. A biomimetic approach for the chemical synthesis of a wide array of 1-substituted tetrahydroisoquinolines was undertaken with the aim of elucidating a common pharmacophore for these compounds with novel mode(s) of anti-TB action.

**Methods:**

Biomimetic Pictet–Spengler or Bischler–Napieralski synthetic routes were employed followed by an evaluation of the biological activity of the synthesized compounds.

**Results:**

In this work, the synthesized tetrahydroisoquinolines were found to inhibit the growth of *M. tuberculosis* H_37_Rv and affect its whole-cell phenotype as well as the activity of the ATP-dependent MurE ligase, a key enzyme involved in the early stage of cell wall peptidoglycan biosynthesis.

**Conclusions:**

As the correlation between the MIC and the half-inhibitory enzymatic concentration was not particularly strong, there is a credible possibility that these compounds have pleiotropic mechanism(s) of action in *M. tuberculosis*.

## Introduction

TB has been a scourge of humanity for millennia and continues to elude many concerted efforts towards its eradication. According to the latest figures from the WHO, 8.6 million people developed the disease in 2012, and 1.3 million people died from it.^1^ Incidences of XDR strains of the TB-causing pathogen *Mycobacterium tuberculosis* are increasing across all inhabited continents and there have been reports of totally drug-resistant strains from India, which has intensified the alarm worldwide.^2^ Infection with these strains is virtually impossible to treat, and particularly in immune-compromised individuals it is almost always a sentence of death.^3^ It is therefore imperative that new anti-TB drugs with novel mode(s) of action are discovered in order to reduce the length of treatment and decimate the resistant strains.

Alkaloids are among the most biologically active plant phytoconstituents. Powerful activities have been described for several alkaloids (tubocurarine, colchicine, camptothecin, quinine and pilocarpine among many others) and some of these scaffolds may still prove useful in new therapeutic areas. Anti-TB alkaloids were the subject of a review in 2009,^4^ and the interest in natural nitrogen-containing compounds for selective TB treatment has since seen a resurgence.^5–8^ The hirsutellone and manzamine classes are some of the most attractive anti-TB alkaloids, displaying high potency and selectivity.^9,10^ The tetrahydroisoquinoline (THI) alkaloid, a noraporphine, extracted from the Colombian tree *Ocotea macrophylla* Kunth, shares structural similarity with other reported anti-TB THIs such as cepharadione B, piperolactam A and (−)-nordicentrine.^11,12^ It has demonstrated antimycobacterial specific activity as well as *M. tuberculosis* MurE (Mtb-MurE) inhibition.^13^

The ATP-dependent Mur ligases (also known as Mur synthetases) are a set of four enzymes Mur C, D, E and F that sequentially add four amino acids: l-alanine (l-Ala), d-glutamic acid (d-Glu), *meso*-diaminopimelic acid (*m*-DAP) and the dipeptide d-alanine-d-alanine (d-Ala-d-Ala) to UDP-MurNAc, resulting in the formation of a precursor of cell wall peptidoglycan (PG). PG is the basal mesh that covalently supports the mycolyl-arabinogalactan unit of the cell wall core; therefore, by targeting the biosynthesis of this macromolecule, the stability of the cell wall would be disrupted, affecting the viability of the mycobacteria.

The genes encoding the Mur synthetases, which are absent in eukaryotic cells, have been demonstrated to be essential for the growth and viability of a wide number of bacterial species^14^ including *M. tuberculosis*^15^ and are conserved in all the bacterial pathogens^16^ including the genetically decayed, obligatory human pathogen *Mycobacterium leprae*.^17^ The Mur synthetases from *M. tuberculosis* have been characterized biochemically and structurally over the past few years by our research group^18–20^ and, through chemical inhibition, have been validated as potential therapeutic targets.^13,21,22^ They contain accessible active sites for drugs or inhibitors to bind to and as yet have not been reported as targets for any of the currently available drugs on the market, making them attractive targets for novel therapeutic entities such as THIs.

THIs are often prepared via the coupling of phenethylamines with carboxylic acids and subsequent Bischler–Napieralski cyclodehydration and reduction.^23^ This strategy, however, requires the protection of hydroxy and amino groups, burdening the synthetic route with rather a large number of steps. We have recently developed biomimetic reaction conditions for the Pictet–Spengler condensation of aldehydes and amines into THIs.^24^ In contrast to the Bischler–Napieralski approach, the biomimetic Pictet–Spengler condensation has only one step and does not require the use of protecting groups. The reaction is mediated by phosphate and is performed under mild reaction conditions (pH 6, 50°C) that are suitable for a variety of less stable or oxidatively sensitive aldehyde and amine substrates.

Building on knowledge of the previously reported THIs, screening the chemical space of substituted THIs could help to elucidate a common anti-TB pharmacophore. The strategy undertaken in this investigation was to screen natural and synthetic 1-substituted isoquinolines against *Mycobacterium bovis* BCG. Active hits were confirmed against *M. tuberculosis* H_37_Rv and their cytotoxicity was surveyed against a murine macrophage (RAW 264.7) cell line. Antibacterial specificity was determined by evaluating the selected compounds against other bacterial species. We previously found that this class of molecules inhibits the MurE ligase of *M. tuberculosis* and therefore their enzymatic inhibitory effect on Mtb-MurE was also explored.

## Materials and methods

### Bacterial strains, cell lines and culture media

The bacterial species used in this study were *M. tuberculosis* H_37_Rv (ATTC 27294), *M. bovis* BCG Pasteur (ATCC 35734), *Mycobacterium aurum* (ATCC 23366), *Mycobacterium smegmatis* mc^2^155 (ATCC 700084), *Rhodococcus equi* RHA1, *Escherichia coli* JM109 (ATCC 53323) and *Pseudomonas putida* (ATCC 12633). The mammalian cell line for assessing cytotoxicity was the murine macrophage cell line RAW264.7 (ATCC TIB-71). Mycobacteria were cultured in Middlebrook 7H9 broth or Middlebrook 7H10 agar media supplemented with albumin/dextrose/catalase (ADC) or oleic acid/albumin/dextrose/catalase (OADC) enrichments, respectively. These supplements were purchased from BD Biosciences. All other bacteria were grown in LB broth or agar. Reagents were purchased from Sigma-Aldrich unless stated otherwise.

### Isolation of (S)-leucoxine

*Rhodostemonodaphne crenaticupula* Madriñan was collected in January 2010 near the Duitama-Charalá road (Department of Boyacá, Colombia). A voucher specimen (COL 544558) was deposited at the Herbario Nacional Colombiano, Universidad Nacional de Colombia. Dried and powdered leaves (810 g) of *R. crenaticupula* were macerated with ethanol at room temperature (25°C). The ethanolic extract was concentrated under reduced pressure (60 g), and fractioned by soxhlet with petroleum ether, chloroform and methanol. The chloroform extract was fractioned by vacuum LC eluting with toluene/isopropyl acetate mixtures (8: 2 to 1: 9) affording 36 fractions. Fractions 3–11 showed a precipitate that was recovered by decantation and purified by Sephadex LH-20 column chromatography eluting with methanol to yield compound **1**. NMR spectra for ^1^H (400 MHz), ^13^C (100 MHz), and 2D [correlation spectroscopy (COSY), heteronuclear multiple bond correlation (HMBC) and heteronuclear multiple quantum correlation (HMQC)] were recorded at 298 K using a Bruker Avance 400 spectrometer. High-resolution MS spectra were acquired using a TOF system (Waters LCT Premier XE) operated in positive mode. Optical rotations were measured using a Bellingham and Stanley ADP440+ digital polarimeter. The detailed spectroscopic data are available as Supplementary data at *JAC* Online.

### Methodology for synthesis

All reagents were obtained from commercial sources and used as received unless otherwise stated. TLC was performed on Kieselgel 60 F_254_ precoated plastic plates, and compounds were visualized by exposure to UV light, potassium permanganate, phosphomolybdic acid or ninhydrin. Flash column chromatography was carried out using silica gel (particle size 40–63 μm). Both analytical and preparative HPLC were performed on a Varian ProStar machine equipped with an autosampler, a UV-visible detector and a Discovery BIO Wide Pore C18-10 Supelco column (25 × 0.46 cm for analytical scale work and 25 × 2.12 cm for preparative scale work). Elutions were monitored at 280 nm and carried out according to either of the following gradients: gradient 1, 5% to 40% of acetonitrile/water (0.1% trifluoroacetic acid or TFA); gradient 2, 5% to 90% of acetonitrile/water (0.1% TFA). NMR spectra for ^1^H and ^13^C were recorded at 298 K at the field indicated using Bruker AMX 300, AMX 400, Avance 500 and Avance 600 machines. Coupling constants were measured in Hertz and referenced to the deuterated solvent used. Infrared spectra were recorded on a PerkinElmer Spectrum 100 FT-IR spectrometer. Optical rotations were recorded on a PerkinElmer model 343 Polarimeter at 589 nm. Mass spectra were recorded on Thermo Finnegan MAT 900XP and Micro Mass Quattro LC electrospray mass spectrometers VG ZAB 2SE. Compounds **2**–**16** were synthesized as previously described.^24^

#### General procedure A (biomimetic Pictet–Spengler reaction)

The amine (1.0 eq.) and aldehyde (1.2 eq.) were added to 10 mL of a 1: 1 mixture of acetonitrile/dipotassium phosphate buffer (0.1 M solution at pH 6). The resulting solution was stirred at 50°C for 12 h. The crude product was concentrated under reduced pressure and purified by preparative HPLC (either gradient 1 or gradient 2), and fractions containing the desired product were combined, concentrated and co-evaporated with methanol (3 × 20 mL).

#### General procedure B (Bischler–Napieralski cyclization and reduction)

The reaction was carried out under anhydrous conditions. Phosphorous oxychloride (10 eq.) and molecular sieves were added to a solution of the amide (1.0 eq.) in anhydrous toluene (10 mL). The reaction mixture was heated at reflux for 2 h, cooled to room temperature (RT) and concentrated under reduced pressure. To the crude material were added NaBH_3_CN (5 eq.), molecular sieves and anhydrous methanol (10 mL), and the reaction was stirred under an inert atmosphere for 2 h. The solvent was then removed *in vacuo* and the product purified using HPLC, either gradient 1 or gradient 2. Fractions containing the desired product were combined, concentrated and co-evaporated with methanol (3 × 20 mL). Detailed synthetic protocols for compounds **17**–**40** and spectroscopic data are available as Supplementary data.

### Evaluation of antibacterial properties

Mycobacterial growth inhibitory activities were examined using the spot culture growth inhibition (SPOTi) assay as previously described.^25,26^ All compounds were dissolved in DMSO at 100 g/L. The assays were performed in a 24-well format in which 2 μL of various dilutions of the compounds were dissolved in 2 mL of Middlebrook 7H10 agar media supplemented with 0.25% glycerol and 10% OADC. A volume of 2 μL of a mid-logarithmic phase culture (∼ 10^6^ cfu/mL) of *M. tuberculosis* H_37_Rv, *M. bovis* BCG Pasteur, *M. aurum* or *M. smegmatis* mc^2^155 grown in Middlebrook 7H9 broth media supplemented with 0.2% glycerol, 0.05% Tween 80 and 10% ADC was added to each well, and the plates were then incubated for 2 weeks at 37°C. The MIC was determined as the minimum concentration of the compounds that completely prevented mycobacterial growth. Isoniazid and rifampicin were used as positive controls and 0.1% DMSO was used as a negative control. The SPOTi assay was performed against *E. coli*, *P. putida* and *R. equi* RHA1 as described, except that LB broth and LB agar were used as growth media. *E. coli* and *P. putida* were grown at 37°C overnight, while *R. equi* RHA1 was grown at 30°C for 48 h.

### Assaying eukaryotic cell toxicity

The murine macrophage cell line RAW264.7 was cultured and maintained as previously described^27^ and the cytotoxicity assay was conducted as previously reported.^28^ Half-growth-inhibitory concentrations (GIC_50_s) were determined by interpolation based on the viability percentage compared with control experiments. Selectivity index (SI) values were calculated by dividing the macrophage GIC_50_ values by the mycobacterial MIC values.

### Pharmacokinetic and pharmacodynamic properties of THIs

Compounds **28** and **35** were assessed on various parameters to determine their potential for progress into further drug development. The following assays were contracted to and performed at Pharmidex, UK and detailed results are provided in the Supplementary data.

#### Human hepatocyte stability assay

The compounds originally dissolved in DMSO were added to PBS to give a resulting concentration of 1 mM. Hepatocytes were then added to the solution and the reaction was incubated at 37°C for up to 120 min. Acetonitrile with internal standard was added to stop the incubation. The samples were centrifuged and the supernatant was then analysed by HPLC-MS/MS to detect the parent compound.

#### Aqueous kinetic solubility

Aqueous kinetic solubility was measured at concentrations of 1 and 0.1 mg/mL. The compounds were equilibrated in 5% DMSO in PBS at 21°C for 24 h. The samples were then centrifuged at 15000 **g** for 10 min. A 100 μL sample of the supernatant was carefully collected and the soluble amount of compound quantified using LC-MS/MS. A standard curve for each compound was prepared in 100% acetonitrile. Solubility results are reported as low (<0.1 mg/mL), medium (0.1–0.5 mg/mL) and high (>0.5 mg/mL).

#### Human microsome stability assay

The compounds originally dissolved in DMSO were added to PBS to give a resulting concentration of 1 mM. Microsomes and NADPH were then added to the solution and the reaction was incubated at 37°C for up to 60 min. Acetonitrile (300 μL) with internal standard was added to stop the incubation. The samples were centrifuged and the supernatant was then analysed by HPLC-MS/MS to detect the parent compound.

#### CYP450 inhibition assay

The compounds originally dissolved in DMSO were added to PBS to give a resulting concentration of 10 mM. CYP baculosomes containing cDNA for a single human P450 isozyme, the fluorogenic substrate and the NADPH were added to the test solution. The reaction was incubated at 37°C for at least 1 h. Fluorogenic substrates metabolized by CYP enzymes result in highly fluorescent products in aqueous solution. The inhibition of CYP prevents this metabolism, which corresponds to a decrease in the fluorescence signal. The fluorescence was measured on a plate reader.

#### Madin–Darby canine kidney (MDCK) cells/cell permeability assay

The compounds were dissolved in 100% DMSO to provide 10 mM stock solutions from which donor (dose) solutions were prepared in DMEM to give a final drug concentration of 10 μM. All dose solutions contained 10 μM propranolol as an internal standard. MDCK cells carrying the *MDR-1* gene were used to seed filters that were exposed to a fixed volume of the donor solution containing the compound of interest. The compound's ability to traverse the monolayer and appear in the receiver compartment was measured over a 30 min period. Bidirectional permeability measurements were derived by examining the transfer of compound from both the apical to the basolateral compartment and vice versa. Sample analysis was conducted using LC-MS/MS with the detection settings optimized for each test compound.

### Purification of recombinant Mtb-MurE and HPLC inhibition assay

The MurE (Rv2158c) protein of *M. tuberculosis* H_37_Rv was overexpressed using a pVLT31 construct on *Pseudomonas putida* KT2442.^18^ A seed culture (10 mL) was prepared in LB broth containing 10 mg/L of rifampicin and 12.5 mg/L of tetracycline. After overnight incubation at 37°C and 200 rpm shaking, the seed culture was used to inoculate 1 L of LB broth containing 12.5 mg/L of tetracycline. The culture was incubated at 30°C and 200 rpm until the OD_600_ reached 0.6, and was then induced with IPTG at a final concentration of 1 mM. The culture was incubated for a further 16 h at 30°C and 200 rpm and then centrifuged at 6000 rpm for 20 min at 4°C. The pellet was resuspended in 20 mL of buffer [20 mM Tris-HCl (pH 8.0)/300 mM NaCl] containing a protease inhibitor cocktail (Roche). The suspension was sonicated on ice (20 cycles of 10 μm amplitude for 45 s with cooling intervals on ice for 30 s) and then centrifuged at 12 000 rpm for 40 min at 4°C. The supernatant was loaded into an Ni^2+^-NTA column equilibrated with buffer and fractions were eluted with buffer containing increasing concentrations of imidazole (25 mM to 200 mM). Pure Mtb-MurE protein was recovered in the 200 mM imidazole fraction as revealed by 12% SDS-PAGE analysis. The recombinant protein was concentrated and imidazole was removed by ultrafiltration with a 10 KDa cut-off size (Spin-X UF, Corning). The HPLC inhibition assay was performed as previously described.^21^ Briefly, the compounds were dissolved in DMSO at a concentration of 25 mM and dilutions were prepared at 8.33 and 2.78 mM. Volumes of 2 μL of these stock compounds and 2 μL of diluted protein (34 mg/L) were added into a 0.5 mL microfuge tube. The substrate mixture was prepared as an aqueous solution containing 25 mM Bis-Tris-propane pH 8.5 buffer, 5 mM MgCl_2_, 100 μM UDP-MurNAc-l-Ala-d-Glu, 250 μM ATP and 1 mM *m*-DAP, and 46 μL of this solution was dispensed into each microfuge tube. The reaction mixtures were incubated at 37°C for 30 min and the protein was then denatured by boiling the mixture at 100°C for 10 min using a heating block. The solutions were centrifuged for 1 min at 4000 rpm and the contents were transferred into 200 μL glass inserts fitted into 2 mL HPLC vials. A mobile phase of 50 mM ammonium formate pH 4.0 was used for the HPLC separation of the substrate and product of the enzymatic reaction eluting at a flow rate of 0.5 mL/min. The column used was an octadecylsilane Jones RP-18 (4.6 mm × 250 mm × 5 mm) plugged to an Agilent 1100 series HPLC machine. The detector used was a diode array system measuring the absorbance at 220 and 268 nm. Controls with and without recombinant enzyme were included.

### Acid-fast staining of M. bovis BCG treated with d-cycloserine and compound 39

Early exponential-phase Middlebrook 7H9 cultures of *M. bovis* BCG (OD_600_ = 0.4) growing in a volume of 100 mL in rolling bottles at 2 rpm and 37°C were treated with 4 × MIC of d-cycloserine (MIC = 25 mg/L) and compound **39** (MIC = 40 mg/L). A control treated with DMSO was included in the experiments. After 2 days of incubation, an aliquot of 100 μL of each sample was dispersed onto a glass slide and dried at 100°C for 10 min. The slide was stained with a Tb-color staining kit (Merck) and observed under a microscope using an oil immersion lens at a magnification of ×1000.

### Field alignment of active molecules

The chemical structures of 3-methoxynordomesticine, leucoxine (**1**), **28** and **35** were drawn in Chemdraw and converted into Mol2 formats. FieldView tool (Cresset software) was used to check the stereochemistry and to calculate the Wildman–Crippen partition coefficient (WCLogP) and total polar surface area. The structures were minimized at a virtual pH of 7.0 and modelled with FieldTemplater (Cresset software) using the default parameters to find the shared three-dimensional patterns of these four molecules based on shape and field similarities.^29^ The high-ranked conformations were used for comparing the similarities between the four molecules.

## Results

### Natural product identification

Compound **1** showed a molecular ion [M+H]^+^ of 356.1498 *m/z* in HRMS, corresponding to a molecular formula of C_20_H_22_NO_5_ (calc. 356.1498). A fragment at 325.1075 *m/z* was observed and attributed to the loss of methylamine, which is a typical feature of aporphine alkaloids with substituents *N*-CH_3_.^30^ Twenty signals appeared in the ^13^C-NMR, and in conjunction with the distortionless enhanced polarization transfer (DEPT) experiments they were assigned to 12 aromatic *sp*^2^ carbons, three methyls, four methylenes and one aliphatic methine at 61.9 ppm. There were 10 quaternary carbons in total. In the ^1^H-NMR spectrum, singlet signals of two aromatic hydrogens were observed at 7.30 (1H, s) and 6.54 (1H, s). In addition, two hydrogens at 6.08 (1H, d, *J* = 1.3 Hz) and 5.92 ppm (1H, d, *J* = 1.3 Hz) correlated in the HMQC spectrum with the signal at 100.6 ppm and were assigned to the methylenedioxy group. Three methyl singlet signals appeared at 3.94, 3.48 and 2.58 ppm, the latter having the typical chemical shift of *N*-CH_3_. The other aliphatic signals had δ ppm values of 3.54 (1H, dd, *J* = 14.4 and 4.6 Hz), 3.19–3.03 (3H, m), 2.63 (1H, dd, *J* = 15.7 and 3.1 Hz), 2.52 (1H, td, *J* = 11.8 and 3.7 Hz) and 2.25 (1H, t, *J* = 14.4 Hz). The spectroscopic experimental data were in complete agreement with the reported data for the known alkaloid leucoxine.^31^ The specific rotation of the aporphine had a positive value ([α]_D_^25 ^= +58.3, *c* 0.09, MeOH), as is the case in aporphines with an (*S*) in configuration atC-6a.^32^ Compound **1** was therefore identified as (*S*)-leucoxine.

### Synthesis

Initially, we employed the biomimetic Pictet–Spengler reaction conditions to synthesize a first generation of over 20 THIs **2**–**24** decorated with a variety of substituents (Table 1) at positions C-1, C-3, C-4, C-6 and C-7 of the THI moiety. The decision was then made to focus on the production of THIs with aromatic or benzylic substituents at C-1 since this feature is common to many bioactive THI natural products such as berberines, aporphines and benzylisoquinoline alkaloids. Most aldehyde and amine substrates required for the reaction were obtained from commercial sources, apart from the phenylacetaldehydes for the synthesis of **2**, **17–21**, which were produced under mild Parikh–Doering oxidation conditions.^24,33^ Preparation of the commercially unavailable amines (for the synthesis of compounds **15** and **16**) was carried out via standard reduction reactions as previously reported.^24,33^ The synthesis of compounds **2**–**16** was performed as previously described using biomimetic Pictet–Spengler condensation,^24^ and **17**–**24** were synthesized using the same method, with a 48%–95% yield. The reaction generally yielded racemic products that were not resolved. However, the condensation of optically active amines or aldehydes resulted in the production of diastereomeric mixtures that could in some cases be separated using preparative HPLC, resulting in the optically enriched compounds indicated (only for compounds **10** and **11**).

**Table 1.** Phosphate-mediated Pictet–Spengler condensation of phenethylamines with aldehydes to give the first-generation THIs^24,42^

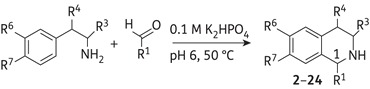						
THI product	R^1^	R^3^	R^4^	R^6^	R^7^	Yield (%)
**2**	CH_2_(4-HOC_6_H_4_)	H	H	OH	OH	63
**3**	CH_2_C_6_H_5_	H	H	OH	OH	73
**4**	2-Pyridyl	H	H	OH	OH	77
**5**	4-MeOC_6_H_4_	H	H	OH	OH	31
**6**	4-HOC_6_H_4_	H	H	OH	OH	35
**7**	3-HOC_6_H_4_	H	H	OH	OH	80
**8**	2-HOC_6_H_4_	H	H	OH	OH	86
**9**	2-Thienyl	H	H	OH	OH	79
**10**	(1*S*)-CH_2_C_6_H_5_	H	(4*R*)-OH	OH	OH	27
**11**	(1*R*)-CH_2_C_6_H_5_	(3*S*)-CO_2_H	H	OH	OH	30
**12**	4-Isoquinolyl	H	H	OH	OH	88
**13**	(1*RS*,2′*S*)-2′,6′-dimethylhept-5′-enyl	H	H	OH	OH	43
**14**	(1*R*,1′*S*)-1′,2′-dihydroxyethyl	H	H	OH	OH	68
**15**	CH_2_C_6_H_5_	H	H	NH_2_	H	52
**16**	CH_2_C_6_H_5_	H	H	OH	H	74
**17**	CH_2_(3-HOC_6_H_4_)	H	H	OH	OH	48
**18**	CH_2_(3,4-MeOC_6_H_3_)	H	H	OH	OH	76
**19**	CH_2_(3-MeO,4-HOC_6_H_3_)	H	H	OH	OH	49
**20**	CH_2_(3,4-OCH_2_OC_6_H_3_)	H	H	OH	OH	80
**21**	CH_2_(2-BrC_6_H_4_)	H	H	OH	OH	95
**22**	1-CH_3_,1-CO_2_H	H	H	OH	OH	52
**23**	cyclohexyl	H	H	OH	OH	91
**24**	C_9_H_19_	H	H	OH	OH	90

Following the MIC data from this first generation of synthetic THIs, a second series of 11 novel compounds **25**–**35** with structures centred on scaffolds possessing 3,4-methylenedioxybenzyl and 2,3-methylenedioxyphenyl moieties were prepared from the corresponding aldehydes **A** and **B** (Table 2). Variations on each of the positions of the THI ring were investigated, with functionalities including methyl, carboxyl, hydroxyl and halogen substituents. The Pictet–Spengler condensation reaction is known to be mainly *para*-directing due to the electron-donating group at C-6. However, this *para*-directed cyclization is mechanistically not possible if a substituent is present at C-9. In this case, and as we observed for **28** and **35**, the cyclization can only proceed *ortho* to the electron-donating group and leads to the 5,8-disubstituted THIs. *Ortho*-cyclization under our biomimetic reaction conditions resulted in the formation of products **28** and **35** at low yields. This is likely to result from steric hindrance around the newly formed carbon–carbon bond as well as competition with undesired side reactions.

**Table 2.** Phosphate-mediated Pictet–Spengler condensation of phenethylamines with aldehydes to give the second series of THIs (**25**–**35**)

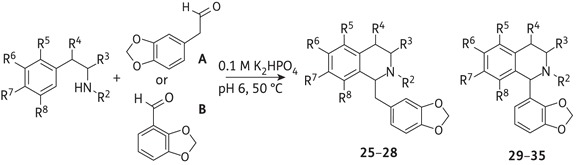									
THI product	Aldehyde used	R^2^	R^3^	R^4^	R^5^	R^6^	R^7^	R^8^	Yield (%)
**25**	A	H	(3*S*)-CH_3_	(4*R*)-OH	H	OH	H	H	93
**26**	A	H	(3*S*)-CO_2_H	H	H	OH	OH	H	96
**27**	A	H	H	H	H	OH	H	H	27
**28**	A	H	H	H	Br	H	H	OH	8
**29**	B	H	H	H	H	OH	OH	H	99
**30**	B	H	H	(4*R*)-OH	H	OH	OH	H	84
**31** (1*R*)	B	CH_3_	H	(4*R*)-OH	H	OH	OH	H	57
**32** (1*S*)	B	CH_3_	H	(4*R*)-OH	H	OH	OH	H	38
**33**	B	H	H	H	H	OH	OH	OH	29
**34**	B	H	H	H	H	OH	H	H	92
**35**	B	H	H	H	Br	H	H	OH	17

**Figure 1.**
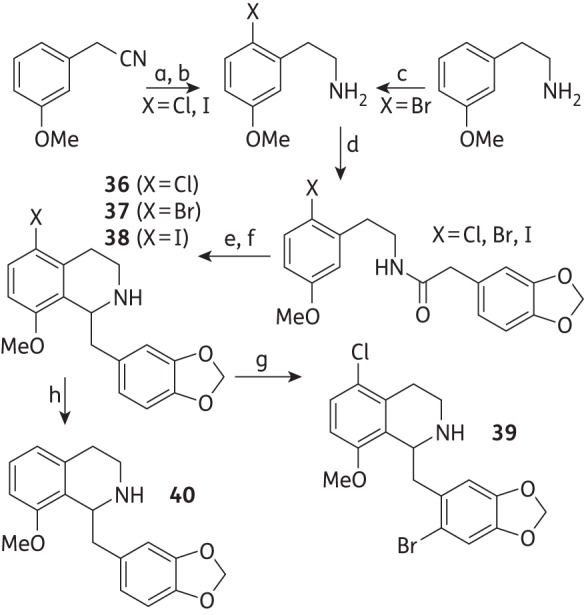
Bischler–Napieralski-mediated synthesis to *ortho*-cyclized THIs **36**–**40**. (a) NCS or NIS, CH_2_Cl_2_; 85% and 81% yield, respectively; (b) For X = Cl and I, B_2_H_6_.SMe_2_, 42% and 55%, respectively. (c) Br_2_, CH_3_CO_2_H, 67%. (d) 3,4-(Methylenedioxy)phenylethanoic acid, *N*,*N*-dicyclohexylcarbodiimide (DCC), 4-dimethylaminopyridine (DMAP), CH_2_Cl_2_; X = Cl and X = Br, 99%, X = I, 74%. (e) POCl_3_, toluene, reflux. (f) NaBH_3_CN, MeOH; **36** 19%, **37** 19%, **38** 2% over two steps. (g) **36**, NBS, CH_2_Cl_2_, 35%. (h) **37**, LiAlH_4_, –78°C, 89%.

The C-5, C-8 disubstituted THIs **28** and **35** displayed good MIC values and prompted us to further investigate close structural analogues of **28**. Since the biomimetic reaction conditions gave the products in low yield, albeit in one step, the alternative Bischler–Napieralski strategy for the synthesis of a third generation of compounds **36**–**40** was investigated (Figure 1 and Table 3).

**Table 3.** Third-generation THIs (**36**–**40**)

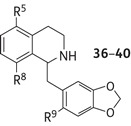			
THI product	R^5^	R^8^	R^9^
**36**	Cl	OMe	H
**37**	Br	OMe	H
**38**	I	OMe	H
**39**	Cl	OMe	Br
**40**	H	OMe	H

Initially, halogenated phenethylamines were prepared via either the chlorination or iodination, using *N*-chlorosuccinimide (NCS) or *N*-iodosuccinimide (NIS), of 3-methoxyphenylacetonitrile, and subsequent reduction using borane. 2-(2-Bromo-5-methoxyphenyl)ethylamine was prepared as previously described.^34^ Coupling with 3,4-(methylenedioxy)phenylacetic acid produced the desired 2-halo-5-hydroxyamides, which were used in *ortho*-directed Bischler–Napieralski condensation reactions to give the corresponding halogenated THIs (**36**, **37** and **38**) in moderate yields. Further modifications included the regioselective bromination of **37** using *N*-bromosuccinimide (NBS) at the C-6′ position of the 3′,4′-methylenedioxylbenzyl substituent, to give **39** in a 35% yield. In addition, reduction of the bromide **37** gave **40** in an 89% yield.

### Anti-TB activity, eukaryotic cell toxicity and pharmacokinetic analysis

Compound **1** was found to inhibit the growth of *M. bovis* BCG and *M. tuberculosis* H_37_Rv with an MIC of 25 and 30 mg/L, respectively (Table 4), and exhibited low cytotoxicity with a GIC_50_ value of 125 mg/L. The SI against *M. tuberculosis* was calculated to be 4.17 (not shown in Table 4), which indicates that it is four times as specific towards the pathogen than it is towards murine macrophage cells.

**Table 4.** MICs for two different species of slow-growing mycobacteria, the GIC_50_ for the murine macrophage cell line RAW264.7 and the SIs of natural and synthetic compounds **1-40**

Name or code	MIC (mg/L) [µM]		GIC_50_ RAW264.7 (mg/L) [µM]	SI (=GIC_50_/MIC)	Mtb-MurE HPLC IC_50_ (µM)
	*M. bovis* BCG	*M. tuberculosis* H_37_Rv			
(*S*)-Leucoxine (**1**)	25 [70]	30 [84]	125 [352]	5.0	820
Norcoclaurine (**2**)	>100 [>369]	ND	425 [1566]	<4.3	837
**3**	>100 [>392]	ND	81 [313]	<0.81	ND
**4**	>100 [>413]	ND	60 [248]	<0.60	ND
**5**	>100 [>369]	ND	91 [335]	<0.91	ND
**6**	>100 [>389]	ND	92 [358]	<0.92	ND
**7**	>100 [>389]	ND	88 [342]	<0.88	ND
**8**	>100 [>389]	ND	87 [338]	<0.87	ND
**9**	>100 [>404]	ND	56 [226]	>0.56	ND
**10**	100 [271]	ND	>250 [>566]	>2.5	ND
**11**	>100 [>334]	ND	87 [291]	<0.87	ND
**12**	>100 [>342]	ND	>250 [>855]	ND	ND
**13**	100 [346]	ND	>250 [>864]	>2.5	ND
**14**	>100 [>444]	ND	85 [377]	<0.85	ND
**15**	>100 [>420]	ND	188 [789]	<1.9	ND
**16**	100 [418]	ND	195 [683]	2.0	ND
**17**	>100 [>369]	ND	64 [236]	<0.64	ND
**18**	>100 [>317]	ND	56 [178]	<0.56	ND
**19**	>100 [>332]	ND	50 [166]	<0.50	ND
**20**	100 [334]	ND	190 [602]	1.9	237
**21**	>100 [>299]	ND	47 [141]	<0.47	ND
**22**	>100 [>448]	ND	178 [797]	<1.8	ND
**23**	>100 [>404]	>100 [>404]	45 [182]	<0.45	ND
**24**	80 [275]	60 [206]	1.6 [5.5]	0.02	186
**25**	>100 [>319]	ND	181 [578]	<1.8	ND
**26**	>100 [>291]	ND	183 [533]	<1.8	ND
**27**	100 [353]	ND	150 [529]	1.5	>1000
**28**	20 [55]	ND	47 [130]	2.3	<111
**29**	>100 [>351]	ND	75 [263]	<0.75	471
**30**	>100 [>332]	ND	89 [295]	<0.89	ND
**31**	>100 [>317]	ND	137 [434]	<1.4	ND
**32**	>100 [>317]	ND	181 [574]	<1.8	ND
**33**	>100 [>332]	ND	5.5 [18]	<0.055	148
**34**	>100 [>371]	ND	181 [672]	<1.8	>1000
**35**	60 [172]	ND	121 [348]	2.0	<111
**36**	40 [121]	60 [181]	41 [124]	1.0	ND
**37**	20 [53]	40 [106]	30 [80]	1.5	165
**38**	50 [118]	30 [71]	28 [66]	0.56	<111
**39**	40 [97]	40 [97]	73 [178]	1.8	ND
**40**	>50 [>168]	>100 [>336]	220 [740]	<4.4	300
Isoniazid	0.1 [0.73]	0.1 [0.73]	3000^a^	30 000	>1000
Rifampicin	0.05 [0.061]	0.05 [0.061]	700^a^	14 000	ND

Enzymatic IC_50_ values for MurE of *M. tuberculosis* were established by HPLC for selected compounds. Isoniazid and rifampicin were included as positive controls. The MIC and IC_50_ of compounds that were not tested are indicated as not determined (ND).

^a^Value taken from Gupta and Bhakta.^27^

**Figure 2.**
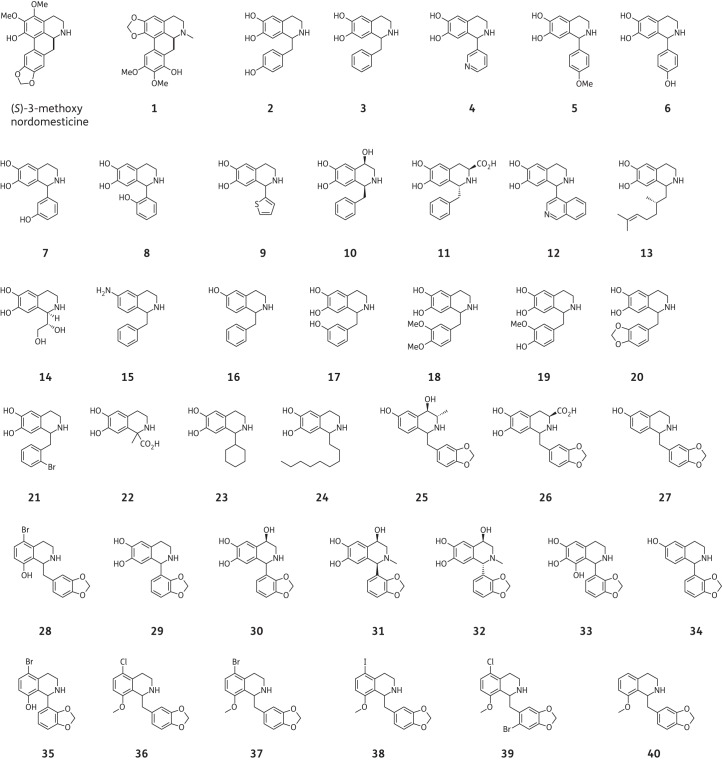
Chemical structures of the natural aporphines 3-methoxynordomesticine and (*S*)-leucoxine (**1**) and the synthetic THIs **2-40**. Compounds **1-40** were evaluated in this study.

The first generation of synthetic 1-substituted THIs (**2–24**) aimed to identify functionalities possessing bioactivity (Figure 2). Norcoclaurine (**2**) did not show significant anti-TB activity (MIC >100 mg/L for *M. bovis* BCG). Most of the compounds from the first generation were also unsuccessful in inhibiting the growth of slow-growing *M. bovis* BCG at 100 mg/L. However, five compounds (**10**, **13**, **16**, **20** and **24**) inhibited the growth of the bacilli with MIC values >60 mg/L. 1-Benzyl-substituted 6,7-dihydroxy THI **10** bearing a hydroxyl group C-4 was active at 100 mg/L and showed low cytotoxicity with a GIC_50_ >250 mg/L. The 1-(1,2-dihydronorgeranyl)-substituted 6,7-dihydroxy THI **13**, 1-benzyl-substituted 6-hydroxy THI **16** and 1-methylenedioxybenzyl-substituted 6,7-dihydroxy THI **20** also displayed an MIC value of 100 mg/L. Moreover, the 1-nonane-substituted 6,7-dihydroxy THI **24** showed an MIC of 80 and 60 mg/L for *M. tuberculosis* H_37_Rv and *M. bovis* BCG, respectively; however, this compound was found to be highly cytotoxic towards macrophages, with a GIC_50_ value of only 1.6 mg/L.

The second-generation compound 3′,4′-methylenedioxybenzyl THI **27**, with a 6-hydroxy substitution, inhibited *M. bovis* BCG at 100 mg/L with moderate cytotoxicity (RAW264.7 GIC_50_ 150 mg/L), achieving an SI value of 1.5. It is noteworthy that the presence of 5-bromo-8-hydroxy substitution (**28** and **35**) conferred a considerable increase in anti-TB potency over the basic THI scaffold. Indeed, 3′,4′-methylenedioxybenzyl **28** and 2′,3′-methylenedioxyphenyl **35** THIs had respective MIC values of 20 and 60 mg/L for *M. bovis* BCG. The SIs were ≥2 for both compounds, suggesting that specificity could be achieved by the 5-bromo-8-hydroxy substitution on the ring. Other THIs from the second generation were less active (MIC >100 mg/L), and surprisingly the 6,7,8-trihydroxy-2′,3′-methylenedioxyphenyl THI **33** was found to be highly toxic to the macrophages, with a GIC_50_ of 5.5 mg/L.

The third generation of THIs (**36–40**) was based on the most selective hit from the second generation, the 5-bromo-8-hydroxy-3′,4′-methylenedioxybenzyl THI **28**. The introduction of a methoxyl substitution to position 8 (in **37**) instead of the phenolic hydroxyl (in **28**) retained the anti-TB activity with an MIC value of 40 and 30 mg/L for *M. tuberculosis* H_37_Rv and *M. bovis* BCG, respectively. However, the compound was much more cytotoxic, with a GIC_50_ of 30 mg/L, and the selectivity was lost. Replacing the 5-bromo substitution of 8-methoxylated **37** for other halogens (chloro in **36** and iodo in **38**) did not change its biological profile, both being cytotoxic. Interestingly, a THI containing a 5′-bromo in ring C but conserving the same substitution of the THI nucleus as in **37** resulted in compound **39**, which was active (MIC 40 mg/L) and less toxic than the parent **37** (Table 3). The **40** derivative bearing both the 8-methoxy and the 3′,4′-methylenedioxybenzyl substitution was inactive, confirming the importance of the halogen substitution on anti-TB activity.

### Antibacterial specificity

As the yield of the best performing THIs (**28** and **35**) was extremely low, three representative compounds (**20**, **24** and **36**) with modest antimycobacterial property and higher yields were evaluated against alternative bacterial species for their antibacterial specificity. Two rapid-growing mycobacterial species, *M. smegmatis* mc^2^155 and *M. aurum*, the acid-fast bacterium *R. equi* RHA1 and two Gram-negative bacteria, *E. coli* and *P. putida*, were screened using the SPOTi assay. The three THIs were completely inactive against both Gram-negative species (MIC >100 mg/L) and the acid-fast bacterium *R. equi* RHA1 (MIC >100 mg/L) (Table 5). THI **20** did not inhibit *M. aurum* or *M. smegmatis* mc^2^155 at 100 mg/L; however, **24** inhibited both *M. aurum* and *M. smegmatis* mc^2^155 at 100 mg/L, and **36** inhibited both rapid-growing mycobacterial species at 60 mg/L.

**Table 5.** MICs of three THIs for two rapid-growing mycobacterial species (*M. aurum* and *M. smegmatis*), an acid-fast bacterium *R. equi* (RHA1) and two Gram-negative bacteria (*E. coli* and *P. putida*)

Compound	MIC (mg/L)				
	*M. aurum*	*M. smegmatis* mc^2^155	*R. equi* RHA1	*E. coli* K12	*P. putida*
**20**	>100	>100	>100	>100	>100
**24**	100	100	>100	>100	>100
**36**	60	60	>100	>100	>100
Rifampicin	0.1	0.1	ND	ND	ND
Kanamycin	ND	ND	1	1	10

ND, not determined.

### Absorption, distribution, metabolism and excretion analysis

Compounds **28** and **35** were selected for pharmacokinetic/pharmacodynamic profiling on the basis of their potent antimycobacterial property and modest SI values (Supplementary data). The hepatocyte stability assay showed both **28** and **35** to have a high hepatic extraction ratios of >0.6. However, the *t*_1/2_ of **28** (23.17 min) in human hepatocytes was much shorter than that of **35** at 78.6 min (Table 6). This trend continued with the microsomal stability studies, with **28** shown to have a *t*_1/2_ of 9.81 min, 10-fold shorter than that of **35**. Both compounds showed encouraging solubility (**35**, 0.3 mg/mL; **28**, 0.8 mg/mL). Membrane permeability revealed compound **35** to be moderately permeable in the MDCK monolayer assay (3.2 × 10^–6^ cm/s) while compound **28** was marginally less permeable (1.7 × 10^–6^ cm/s). Inspection of the efflux permeability ratios suggested that both compounds were P-glycoprotein substrates, and metabolic turnover in both microsomes and hepatocytes confirmed both compounds to be highly metabolized.

**Table 6.** Microsomal and hepatocytic stability of compounds **28** and **35**

Compound	*t*_1/2_ (min)		CL_int_ (µL/min/million cells)
	microsomes	hepatocytes	
**28**	9.8	23.2	17.4
**35**	109.6	78.6	12.6

The *t*_1/2_ of both compounds in hepatocytes and microsomes was determined and their *in vivo* hepatic intrinsic clearance (CL_int_) prediction values are shown.

### MurE synthetase inhibitory properties

Thirteen compounds were tested for their enzymatic inhibition of the MurE synthetase of *M. tuberculosis* using the previously reported HPLC assay.^21^ Isoniazid was employed as a negative control, showing no inhibitory effect at any of the concentrations examined. IC_50_ values were calculated by interpolation of the discrete inhibitory percentage data based on the AUCs of the UDP-MurNAc-l-Ala-γ-d-Glu-*m*-DAP product (Table 4). Three compounds (**28**, **35** and **38**) demonstrated particularly effective MurE inhibition, with IC_50_ values <111 μM. Six others (**20, 24, 29, 33, 37** and **40**) had a range of IC_50_ values between 111 μM and 500 μM, while the natural product (*S*)-leucoxine (**1**) and synthetic norcoclaurine (**2**) showed IC_50_ values ∼830 μM. Only two THIs exhibited no enzymatic inhibitory effect (IC_50_ > 1 mM). Interestingly, compounds bearing a halogen atom at C-5 of the THI nucleus such as **28**, **35**, **37** and **38** were all inhibitory to the MurE ligase, with IC_50_ < 200 μM. A positive trend (*r* = 0.549) between the enzymatic inhibition of Mtb-MurE (IC_50_) and the growth inhibition of *M. tuberculosis* H_37_Rv (MIC) was observed for eight THIs, which had IC_50_ and MIC values in the range 40–400 μM. The linear fit of the experimental data had a slope of 0.544 and a *y*-intercept of 92.12 µM. The *t*-test reported *P *< 0.1, indicating that there was a weak relationship between the two variables.

### Field alignment of active molecules

**Figure 3.**
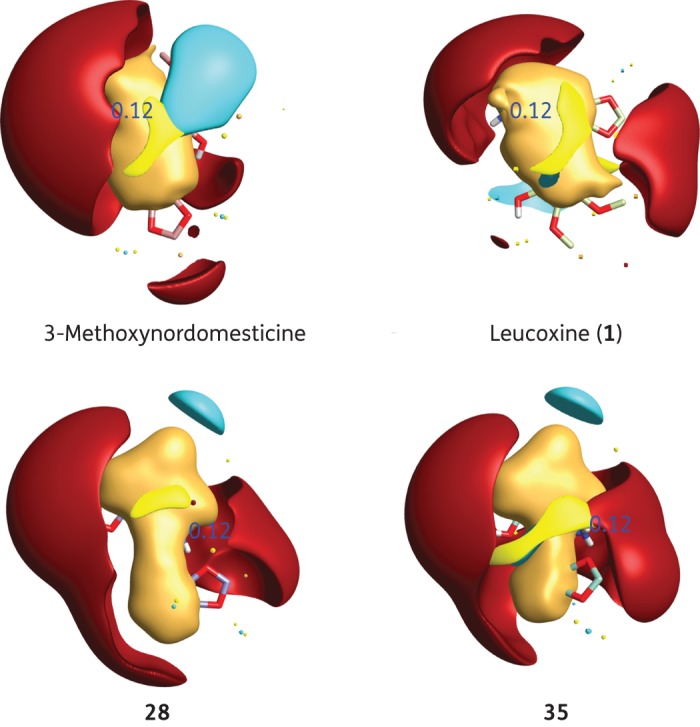
Comparison of the three-dimensional surface of four active alkaloids using FieldTemplater (Cresset software). Alignment of 3-methoxynordomesticine, leucoxine (**1**), **28** and **35** showing surfaces of different chemical environments. Turquoise surfaces represent negative patches, red surfaces correspond to positive sections, brick yellow surfaces denote lipophilic regions and light yellow volumes relate to dominant van der Waals areas. This figure appears in colour in the online version of *JAC* and in black and white in the print version of *JAC*.

The alignment of 3-methoxynordomesticine, leucoxine (**1**), **28** and **35** showed that the four molecules generated a similar three-dimensional distribution of the electrostatic, lipophilic and van der Waals fields (Figure 3). Even in cases where the chemical groups did not exactly match [for instance, the methylenedioxy substituted ring is oriented in a different direction in leucoxine (**1**) compared with 3-methoxynordomesticine, **28** and **35**], the fields generated were spatially concurrent. The score obtained in the alignment of these four molecules (FieldTemplater global score 0.689) suggested that these four molecules created a similar three-dimensional environment around them.

### Phenotype analysis

**Figure 4.**
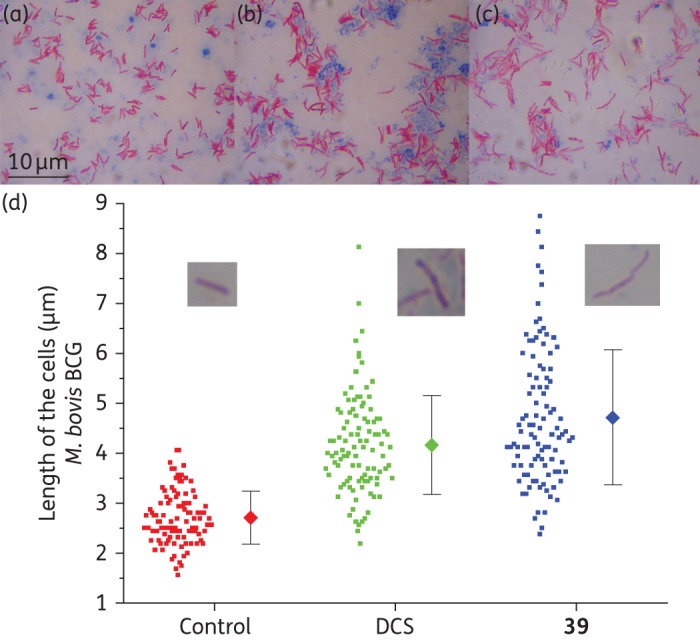
Acid fast staining of *M. bovis* BCG after 48 h of treatment with d-cycloserine (DCS) and **39**, and comparison of the cell length. (a) Control with DMSO. (b) DCS at 100 mg/L. (c) Compound **39** at 160 mg/L. Magnification ×1000. (d) The cell length of *M. bovis* BCG after treatment with vehicle (DMSO), DCS (100 mg/L) and **39** (160 mg/L). The dots represent the raw data and the diamonds represent the mean value with SD (error bars). The cell length of 100 cells was manually measured for each treatment from microscopic images. The mean cell sizes of the three treatments were statistically significant at *P* < 0.01. This figure appears in colour in the online version of *JAC* and in black and white in the print version of *JAC*.

Liquid *M. bovis* BCG cultures treated at an early exponential phase with 4 × MIC concentrations of d-cycloserine (100 mg/L) and of **39** (160 mg/L) showed a distinct cellular phenotype in the microscopic acid-fast staining observation (Figure 4). Cells treated with either d-cycloserine or **39** were significantly longer than the control treated with the vehicle (DMSO). The untreated control showed a cell length of 2.7 ± 0.5 μm, while cells treated with d-cycloserine had a much longer length of 4.2 ± 1.0 μm. A similar elongation was observed with **39**, showing a length of 4.7 ± 1.4 μm. The mean cell sizes of the three treatments were statistically significant with *P *< 0.01 by analysis of variance.

## Discussion

(*S*)-Leucoxine (**1**) isolated from *Rhodostemonodaphne crenaticupula* Madriñan was found to completely inhibit the growth of *M. bovis* BCG and *M. tuberculosis* H_37_Rv at around 25–30 mg/L. The substitution of this aporphine alkaloid was similar to that of the previously reported hit 3-methoxynordomesticine.^13^ However, **1** was less cytotoxic with an SI of 4 while the SI of 3-methoxynordomesticine was around 1. This result indicated that interchanging the chemical substitutions from rings A to D had an insignificant effect on the compound's anti-TB activity but a considerable effect on the eukaryotic cell toxicity. (-)-Nordicentrine, which has a methylenedioxy group on ring A and a dimethoxy substitution on ring D, has been reported to have an MIC value of 12.5 mg/L for the virulent *M. tuberculosis* H_37_Rv strain, also being cytotoxic against cancer cell lines.^12^

Among the first generation of THIs (**2–24**), several interesting structure–activity relationships were extracted from the whole-cell biological data. By comparing the structures and MIC values of **3** and **16**, it was noted that a single hydroxyl at C-6 (as in **16**) resulted in inhibition at 100 mg/L while the presence of a dihydroxy substitution at C-6 and C-7 (as in **3**) did not lead to inhibition at the same concentration. The presence of a hydroxyl group at C-4 of the 6,7-dihydroxy-substituted-THI nucleus in compound **10** also conferred growth-inhibitory activity at 100 mg/L. Moreover, the presence of a lipophilic chain at C-1 of the 6,7-dihydroxy substituted-THIs (in **13** and **24**) showed a tendency for an increase in the mycobacterial inhibitory properties. A differential effect in terms of cytotoxicity was observed between these compounds, with the 1,2-dihydronorgeranyl-substituted THI (**13**) being far less cytotoxic than the nonane-substituted THI (**24**), perhaps due to the greater surfactant-like properties of **24**. It was interesting to observe that the 1-(3′,4′-methylenedioxybenzyl)-6,7-dihydroxy THI (**20**), which is the compound most structurally similar to 3-methoxynordomesticine, was able to inhibit mycobacterial growth at 100 mg/L, suggesting that the 3′,4′-methylenedioxybenzyl substitution conferred anti-TB activity compared with other benzyl substitutions.

The second generation of compounds (**25-35**) shared either the 3′,4′-methylenedioxybenzyl substitution (**25-28**) or the 2′,3′-methylenedioxyphenyl substitution (**29-35**). As mentioned earlier, the 5-bromo-8-hydroxy substitution (in both **28** and **35**) undoubtedly conferred marked anti-TB activity. It still remains to be fully elucidated whether the halogen or the hydroxyl substitution is responsible for the activity of these entities, as removal of the halogen or methylation of the hydroxyl in the third-generation compounds led to lower anti-TB activities. The benzyl derivative (**28**) was three times more active than the phenyl derivative (**35**), probably because of the increased conformational flexibility of the former, which could influence binding to the molecular target. In general, the benzylic derivatives seem more promising than the phenyl derivatives. As observed for **16**, the single phenolic group at C-6 of **27** was deemed important for retaining the anti-TB activity, although derivatization at this position has yet to be explored.

Pharmacokinetic/pharmacodynamic analyses showed that **28** had a high aqueous solubility, probably translating to a more hydrophilic nature, which would explain its lower cellular permeability compared with **35**. In addition, **28** demonstrated higher instability in both hepatocytes and microsomes, meaning that it was metabolized rapidly and had low retention times. It also inhibited the activity of CYP450 enzymes to a greater extent than **35**. As both **28** and **35** were found to be substrates for P-glycoprotein efflux mechanisms, the use of these compounds in combination with existing, potent efflux pump inhibitors could result in a significantly enhancement of their anti-TB properties.

The study of anti-TB properties of the third generation (**36**–**40**) of compounds indicated that the presence of bromine at C-5 led to a greater inhibitory potency compared with the chloro- or iodo-analogues. It is probable that size and the electronegativity of the bromine atom contribute favourably to optimal contact with the target macromolecule. In addition, when comparing **36**–**38** with **35**, it was clear that the 8-methoxy derivatives were much more toxic than the 8-hydroxy compound **35** (without a significant gain in antimycobacterial activity). Interestingly, a further substitution with a bromo group at 2′ on the benzyl ring reduced the cytotoxicity while maintaining effective mycobacterial killing. This is possibly due to conformational or steric effects induced by the presence of a halogen.^35^

The synthetic THIs were equally active against other environmental species of mycobacteria such as *M. smegmatis* and *M. aurum*; however, they were not growth inhibitors of Gram-negative bacteria such as *E. coli* or *P. putida*, or acid-fast *Rhodococcus equi* RHA1. The mycobacterial specificity of this class was previously demonstrated by us for 3-methoxynordomesticine^13^ and seems to suggest that the class inhibits a specific endogenous mechanism particular to *Mycobacterium*.

Among the 13 compounds examined for inhibition of Mtb-MurE activity, 11 compounds showed enzymatic inhibition, three of which had IC_50_ values <111 μM. It seems reasonable that the aporphines^13^ and THIs share a common pharmacophore, which inhibits Mtb-MurE ligase. According to the computational field alignment of the molecules, the key structural motif seems to be the presence of dissimilar pairs of electrostatically positive patches (in red in Figure 3), caused by the nitrogen atoms and the relative spatial closeness of the methylenedioxy groups. The presence of halogens in position 5 of the isoquinoline nucleus clearly increased Mtb-MurE inhibitory activity. For instance, **40** showed an Mtb-MurE IC_50_ value of 300 μM whereas those compounds with halogens displayed an Mtb-MurE IC_50_ value <170 μM. The effect appears to be more pronounced with larger halogens such as bromine and iodine (**35** and **38**) in comparison with chlorine (**36**). A phenolic substitution in position 8 of the isoquinoline nucleus also conferred increased Mtb-MurE inhibition (as seen by a comparison of the Mtb-MurE IC_50_ for compounds **29** and **33**).

There is a positive trend between the inhibition of Mtb-MurE (IC_50_) and the anti-TB activity (MIC), as evidenced by the plot correlating the two variables. An increase in Mtb-MurE IC_50_ is associated with an increase in the whole-cell MIC, suggesting that MurE inhibition contributes to the antimicrobial effect observed for this class. However, the correlation is weak as the *t*-test suggests there is 1 in 10 probability that this observation occurs by chance. This potentially indicates that there could be other potential molecular targets involved in the phenotypic inhibitory effect of the THIs.

Mycobacterial cells treated with 4 × MIC concentrations of compound **39** or d-cycloserine showed significant morphological cellular elongation when compared with the control treatment (Figure 4). It is well known that d-cycloserine inhibits both d-alanine racemase and d-alanine:d-alanine ligase in *M. tuberculosis*,^36,37^ essential enzymes for the biosynthesis of the d-alanine optical isomer and its dimer, which are ultimately required for building the cytoplasmic PG precursor UDP-MurNAc-l-Ala-γ-d-Glu-m-DAP-d-Ala-d-Ala. Treatment with other inhibitors of PG biosynthesis such as vancomycin and cephalosporin C also results in elongated cells (J. D. Guzman, A. Maitra and S. Bhakta, unpublished data). This elongated phenotype has been observed in mycobacterial cells overexpressing or depleting serine/threonine kinases PknA/B^38^ or overexpressing the cell-division suppressor Rv2719c,^39^ proteins that are associated with the regulation of PG biosynthesis and recycling. It is well established that filamentous-shaped bacteria (another term for elongated cells) typically appear as a consequence of an interruption in cell division^40^ and it is therefore not surprising that drugs affecting PG biosynthesis provoke this phenotype. Although drugs targeting other pathways can also arrest cell division and lead to cell elongation (for instance, quinolones also elongate the cells),^40,41^ the observed phenotype is consistent with an unbalanced cell wall assembly, a feature that could be explained by an inhibition of ATP-dependent Mur ligases. This hypothesis is favoured by our experimental data; however, further investigation of the molecular mechanism(s) of action is needed, which may ultimately boost chemical design efforts to develop a successful anti-TB scaffold.

## Funding

This work was supported by the Medical Research Council, UK (MRC New Investigators Research Grant, code: G0801956 to S. B.) and the Biotechnology and Biological Sciences Research Council (BBSRC code: BB/G014426/1 to T. P. and M. C. G.). GlaxoSmithKline and the Engineering and Physical Sciences Research Council (EPSRC) provided PhD funding for E. D. L. and Bloomsbury Colleges, University of London provided PhD funding for J. D. G. The funders, including GlaxoSmithKline, had no role in study design, data collection and analysis, decision to publish or preparation of the manuscript.

## Transparency declarations

J. M. R. and D. N. are employees of GlaxoSmithKline and both own small numbers of GlaxoSmithKline shares. All other authors, none to declare.

## Supplementary data

Supplementary data are available at *JAC* Online (http://jac.oxfordjournals.org/).

Supplementary Data
