# Supplementary material for: Tetrahydroisoquinolines affect the whole-cell phenotype of Mycobacterium tuberculosis by inhibiting the ATP-dependent MurE ligase
Source: J Antimicrob Chemother. 2015 Feb 4;70(6):1691–703. doi: 10.1093/jac/dkv010 (PMC4498294; doi:10.1093/jac/dkv010)
Supplement: Supplementary Data [file supp_70_6_1691__index.html]

Tetrahydroisoquinolines affect the whole-cell phenotype of Mycobacterium tuberculosis by inhibiting the ATP-dependent MurE ligase — Tetrahydroisoquinolines affect the whole-cell phenotype of Mycobacterium tuberculosis by inhibiting the ATP-dependent MurE ligase — Supplementary Data 

# Tetrahydroisoquinolines affect the whole-cell phenotype of *Mycobacterium tuberculosis* by inhibiting the ATP-dependent MurE ligase

## Supplementary Data

Supplementary Data

**Files in this Data Supplement:**

- Supplementary data\_I - docx file
- Supplementary data\_II - xlsx file
