# Supplementary material for: Tetrahydroisoquinolines affect the whole-cell phenotype of Mycobacterium tuberculosis by inhibiting the ATP-dependent MurE ligase
Source: J Antimicrob Chemother. 2015 Feb 4;70(6):1691–703. doi: 10.1093/jac/dkv010 (PMC4498294; doi:10.1093/jac/dkv010)
Supplement: Supplementary Data [file supp_dkv010_dkv010supp_data_I.docx]

**Supplementary data**

**(S)-Leucoxine (1)**

^1^H NMR (400 MHz; CDCl_3_) δ 2.25 (1H, t, *J* = 14.4 Hz, 7-*H*H), 2.52 (1H, td, *J =* 11.8 and 3.7 Hz, 5-*H*H), 2,59 (3H, s, 12-CH_3_), 2.63 (1H, dd, *J =* 15.7 and 3.1 Hz, 4-*H*H), 3.19-3.03 (3H, m, 4-H*H*, 5-H*H*, 6-*H*H), 3.54 (1H, dd, *J* = 14.4 and 4.6 Hz, 7-H*H*), 3.90 (3H, s, 9-OCH_3_), 3.94 (3H, s, 10-OCH_3_), 5.92 (1H, d, *J =* 1.3 Hz, OC*H*HO), 6.08 (1H, d, *J =* 1.3 Hz, OC*H*HO), 6.54 (1H, s, 3-H), 7.31 (1H, s, 11-H); ^13^C NMR (100 MHz; CDCl_3_) δ 25.9, 29.2, 43.9, 53.6, 55.9, 61.0, 62.0, 100.6, 103.1, 107.3, 114.7, 116.5, 126.7, 126.9, 131.9, 134.9, 142.2, 146.0, 146.6, 150.5;*m/z* [HRMS ES+] found [MH]^+^ 356.1498. C_20_H_22_NO_5_ requires 356.1498.

**1-(3-Hydroxybenzyl)-1,2,3,4-tetrahydroisoquinoline-6,7-diol** (**17).** Compound **17** was prepared according to procedure **A** using dopamine hydrochloride (57 mg, 0.30 mmol) and (3-hydroxyphenyl)acetaldehyde) [(Pesnot, et al., 2012)](#_ENREF_33) (41 mg, 0.30 mmol). The product was purified by preparative HPLC (gradient **1**, retention time 13.5 min) to give **17** as a colourless oil (39 mg, 48%). ^1^H NMR (600 MHz; CD_3_OD) δ 2.86–3.04 (3H, m, 4-H_2_, NCHC*H*H), 3.25 (1H, app. quint, *J* = 6.3 Hz, 3-*H*H), 3.39 (1H, dd, *J* = 14.5 and 5.6 Hz, NCHCH*H*), 3.45 (1H, app. quint, *J* = 6.3 Hz, 3-H*H*), 4.62 (1H, dd, *J* = 8.6 and 5.6 Hz, 1-H), 6.61‒6.63 (2H, m, 5-H and 8-H)*,* 6.74‒6.77 (2H, m, 2'-H and 4'-H), 6.79 (1H, d, *J* = 7.9 Hz, 6'-H), 7.20 (1H, t, *J* = 7.9 Hz, 5'-H); ^13^C NMR (150 MHz; CD_3_OD) δ 25.7, 41.0, 41.2, 57.7, 114.1, 115.7, 116.2, 117.1, 121.5, 123.67, 123.70, 131.3, 138.1, 145.8, 146.9, 159.3; *m/z* [ES+] 272 (MH^+^, 30%), 255 (100) and 164 (58);  *m/z* [HRMS ES+] found [MH]^+^ 272.1280. C_16_H_18_NO_3_ requires 272.1287.

**1-(3,4-Dimethoxybenzyl)-1,2,3,4-tetrahydroisoquinoline-6,7-diol** (**18)** Compound **18** was prepared according to procedure **A** using dopamine hydrochloride (76 mg, 0.40 mmol) and (3,4-dimethoxyphenyl)acetaldehyde [(Pesnot, et al., 2012)](#_ENREF_33) (72 mg, 0.40 mmol). The product was purified by preparative HPLC (gradient **1**, retention time 15.0 min) to give **18** [(Ruff, et al., 2012)](#_ENREF_42) as a colourless oil (96 mg, 80%). ^1^H NMR (600 MHz; CD_3_OD) δ 2.87–3.06 (3H, m, 4-H_2_, NCHC*H*H), 3.25 (1H, app. quint, *J* = 6.3 Hz, 3-*H*H), 3.39 (1H, dd, *J* = 14.5 and 5.9 Hz, NCHCH*H*), 3.44 (1H, app. quint, *J* = 6.3 Hz, 3-H*H*), 3.80 (3H, s, OCH_3_), 3.82 (3H, s, OCH_3_), 4.63 (1H, dd, *J* = 8.0 and 5.9 Hz, 1-H), 6.61 (1H, s, 8-H)*,* 6.63 (1H, s, 5-H)*,* 6.84‒6.88 (2H, m, 2'-H and 6'-H), 6.94 (1H, d, *J* = 7.9 Hz, 5'-H); ^13^C NMR (150 MHz; CD_3_OD) δ 25.7, 40.8, 40.9, 56.38, 56.43, 57.6, 113.3, 114.1, 114.3, 116.2, 123.1, 123.6, 123.7, 129.1, 145.8, 146.7, 150.1, 150.8; *m/z* [HRMS ES+] found [MH]^+^ 316.1563. C_18_H_22_NO_4_ requires 316.1549.

**1-(4-Hydroxy-3-methoxybenzyl)-1,2,3,4-tetrahydroisoquinoline-6,7-diol** (**19).** Compound **19** was prepared according to procedure **A** using dopamine hydrochloride (76 mg, 0.40 mmol) and (4-hydroxy-3-methoxyphenyl)acetaldehyde [(Pesnot, et al., 2012)](#_ENREF_33) (66 mg, 0.40 mmol). The product was purified by preparative HPLC (gradient **1**, retention time 13.0 min) to give **19** [(Brossi, et al., 1980)](#_ENREF_43) as a colourless oil (59 mg, 49%). ^1^H NMR (600 MHz; CD_3_OD) δ 2.87–3.03 (3H, m, 4-H_2_, NCHC*H*H), 3.24 (1H, app. quint, *J* = 6.3 Hz, 3-*H*H), 3.38 (1H, dd, *J* = 14.5 and 5.7 Hz, NCHCH*H*), 3.44 (1H, app. quint, *J* = 6.3 Hz, 3-H*H*), 3.83 (3H, s, OCH_3_), 4.61 (1H, dd, *J* = 8.5 and 5.7 Hz, 1-H), 6.63 (1H, s, 5-H)*,* 6.64 (1H, s, 8-H)*,* 6.75 (1H, dd, *J* = 8.0 and 1.6 Hz, 6'-H), 6.81 (1H, d, *J* = 8.0 Hz, 5'-H), 6.83 (1H, d, *J* = 1.6 Hz, 2'-H); ^13^C NMR (150 MHz; CD_3_OD) δ 25.8, 40.8, 40.9, 56.3, 57.8, 113.8, 114.2, 116.2, 116.7, 123.2, 123.66, 123.69, 127.6, 145.8, 146.9, 147.3, 149.5; *m/z* [HRMS ES+] found [MH]^+^ 302.1387. C_17_H_20_NO_4_ requires 302.1392.

**1-(1,3-Benzodioxol-5-ylmethyl)-1,2,3,4-tetrahydroisoquinoline-6,7-diol** (**20).** Compound **20** was prepared according to procedure **A** using dopamine hydrochloride (57 mg, 0.30 mmol) and 1,3-benzodioxole-5-acetaldehyde [(Pesnot, et al., 2012)](#_ENREF_33) (50 mg, 0.30 mmol). The product was purified by preparative HPLC (gradient **1**, retention time 15.0 min) to give **20** [(Schöpf and Salzer, 1940)](#_ENREF_44) as a colourless oil (72 mg, 80%). ^1^H NMR (600 MHz; CD_3_OD) δ 2.87–3.03 (3H, m, 4-H_2_, NCHC*H*H), 3.25 (1H, app. quint, *J* = 6.4 Hz, 3-*H*H), 3.38 (1H, dd, *J* = 14.4 and 5.4 Hz, NCHCH*H*), 3.47 (1H, app. quint, *J* = 6.4 Hz, 3-H*H*), 4.59 (1H, dd, *J* = 8.4 and 5.4 Hz, 1-H), 5.96 (2H, s, OCH_2_O), 6.62 (1H, s, 8-H)*,* 6.63 (1H, s, 5-H)*,* 6.77 (1H, dd, *J* = 7.8 and 1.2 Hz, 6'-H), 6.82 (1H, d, *J* = 7.8 Hz, 5'-H), 6.84 (1H, d, *J* = 1.2 Hz, 2'-H); ^13^C NMR (150 MHz; CD_3_OD) δ 25.7, 40.8, 40.9, 57.8, 102.6, 109.7, 110.5, 114.1, 116.2, 123.6, 123.7, 123.9, 130.1, 145.8, 146.9, 148.7, 149.8; *m/z* [HRMS CI+] found [MH]^+^ 300.12369. C_17_H_18_NO_4_ requires 300.12358.

**(2-Bromophenyl)acetaldehyde.** The reaction was carried out under anhydrous conditions. To a solution of 2-(4-bromophenyl) ethanol (2.00 g, 9.95 mmol) in DMSO/CH_2_Cl_2_ (15 mL) was added *N*,*N*-diisopropylethylamine (2.5 eq.) followed by the dropwise addition of a solution of SO_3_.pyridine (2.5 eq.) in DMSO/CH_2_Cl_2_ (10 mL) over 30 min at -15 °C. The mixture was stirred for 1 h at -15 °C and quenched by the addition of ice-cold water (50 mL). The aqueous layer was extracted with CH_2_Cl_2_ (3 × 50 mL) and the organic layers combined and concentrated under reduced pressure. The crude material was purified using flash silica chromatography (5% EtOAc in hexane) to give the titled compound[(Hartman, et al., 1985)](#_ENREF_45) as a pale yellow oil (780 mg, 39%). ^1^H NMR (600 MHz; CDCl_3_) δ 3.87 (2H, d, *J* = 1.6 Hz, CH_2_), 7.19 (1H, td, *J* = 7.6 and 1.4 Hz, 4-H), 7.24 (1H, dd, *J* = 7.6 and 1.4 Hz, 6-H), 7.32 (1H, t, *J* = 7.6 Hz, 5-H), 7.62 (1H, d, *J* = 7.6 Hz, 3-H), 9.74 (1H, t, *J* = 1.6 Hz, CHO); ^13^C NMR (150 MHz; CDCl_3_) δ 50.6, 125.0, 127.9, 129.3, 131.8, 132.6, 133.1, 198.4; *m/z* [HRMS EI] found M^+^ 197.96767. C_8_H_7_^79^BrO requires 197.96747.

**1-(2-Bromobenzyl)-1,2,3,4-tetrahydroisoquinoline-6,7-diol** (**21).** Compound **21** was prepared according to procedure **A** using dopamine hydrochloride (100 mg, 0.53 mmol) and (2-bromophenyl)acetaldehyde [(Hartman, et al., 1985)](#_ENREF_45) (115 mg, 0.58 mmol). The product was purified by preparative HPLC (gradient **2**, retention time 16.0 min) to give **21** as a colourless oil (168 mg, 95%). ^1^H NMR (600 MHz; CD_3_OD) δ 2.90–3.09 (2H, m, 4-H_2_), 3.21 (1H, dd, *J* = 14.3 and 8.8 Hz, NCHC*H*H), 3.27‒3.33 (1H, m, 3-*H*H), 3.56‒3.63 (2H, m, 3-H*H* and NCHCH*H*), 4.73 (1H, dd, *J* = 8.8 and 6.0 Hz, 1-H), 6.51 (1H, s, 8-H)*,* 6.64 (1H, s, 5-H)*,* 7.26 (1H, td, *J* = 7.6 and 1.4 Hz, 4'-H), 7.32 (1H, dd, *J* = 7.3 and 1.4 Hz, 6'-H), 7.36 (1H, app. t, *J* = 7.4 Hz, 5'-H), 7.67 (1H, d, *J* = 7.6 Hz, 3'-H); ^13^C NMR (150 MHz; CD_3_OD) δ 25.6, 40.8, 41.8, 55.9, 114.2, 116.3, 123.1, 123.8, 126.0, 129.4, 130.9, 133.5, 134.4, 136.0, 145.8, 147.0; *m/z* [ES+] 336 (M[^81^Br]H^+^, 94%), 334 (M[^79^Br]H^+^, 92), 319 (96), 317 (100), 212 (45), 210 (35); *m/z* [HRMS ES+] found [MH]^+^ 334.0434. C_16_H_17_NO_2_^79^Br requires 334.0443.

**1-Isoquinolinecarboxylic acid-1,2,3,4-tetrahydro-6,7-dihydroxy-1-methyl (22).** Compound **22** was prepared according to procedure **A** using dopamine hydrochloride (100 mg, 0.53 mmol) and pyruvic acid (56 mg, 0.64 mmol). The product was purified by preparative HPLC (gradient **1**, retention time 13.5 min) to give **22**[(Hahn and Stiehl, 1936)](#_ENREF_46) as a colourless oil (61 mg, 52%). ^1^H NMR (600 MHz; D_2_O) δ 1.88 (3H, s, CH_3_), 2.88–3.03 (2H, m, 4-H_2_), 3.49‒3.55 (2H, m, 3-H_2_), 6.73 (1H, s, 5-H)*,* 7.09 (1H, s, 8-H); ^13^C NMR (150 MHz; D_2_O) δ 24.7, 24.8, 39.9, 63.4, 114.9, 116.2, 124.2, 124.5, 143.8, 145.2, 173.9; *m/z* [HRMS ES+] found [MH]^+^ 224.0925. C_11_H_14_NO_4_ requires 224.0923.

**1-Cyclohexyl-1,2,3,4-tetrahydroisoquinoline-6,7-diol** (**23).** Compound **23** was prepared according to procedure **A** using dopamine hydrochloride (100 mg, 0.53 mmol) and cyclohexanecarboxaldehyde (88 mg, 0.78 mmol). The product was purified by preparative HPLC (gradient **1**, retention time 15.5 min) to give **23** as a colourless oil (126 mg, 96%). *ν*_max_ (neat)/cm^–1^ 3150, 2933, 2857, 1609, 1527; ^1^H NMR (600 MHz; CD_3_OD) δ 1.04–1.90 (10H, m, 5 × CH_2_-cyclohexyl), 2.05 (1H, m, NCHC*H*), 2.82‒3.02 (2H, m, 4-H_2_), 3.23‒3.29 (1H, m, 3-*H*H), 3.49‒3.56 (1H, m, 3-H*H*), 4.25 (1H, d, *J* = 5.2 Hz, 1-H), 6.61 (1H, s, 5-H)*,* 6.65 (1H, s, 8-H); ^13^C NMR (150 MHz; CD_3_OD) δ 25.8, 27.10, 27.12, 27.4, 27.7, 31.0, 41.6, 42.0, 61.5, 114.3, 116.3, 122.8, 124.5, 145.8, 146.7; *m/z* [HRMS ES+] found [MH]^+^ 248.1640. C_15_H_22_NO_2_ requires 248.1651.

**1-Nonyl-1,2,3,4-tetrahydroisoquinoline-6,7-diol** (**24).** Compound **24** was prepared according to procedure **A** using dopamine hydrochloride (100 mg, 0.53 mmol) and decanal (123 mg, 0.79 mmol). The product was purified by preparative HPLC (gradient 30-60% acetonitrile in water gradient [with 0.1% TFA], retention time 15.0 min) to give **24** as a colourless oil (138 mg, 89%). *ν*_max_ (neat)/cm^–1^ 3463, 3063, 2922, 1616; ^1^H NMR (600 MHz; CD_3_OD) δ 0.88 (3H, t, *J* = 6.6 Hz, CH_3_), 1.25–52 (14H, m, NCHCH_2_(C*H*_2_)_7_CH_3_), 1.84‒2.05 (2H, m, NCHC*H*_2_), 2.86‒3.01 (2H, m, 4-H_2_), 3.28‒3.56 (2H, m, 3-H_2_), 4.33 (1H, dd, *J* = 7.9 and 5.0 Hz, 1-H), 6.61 (1H, s, 5-H)*,* 6.65 (1H, s, 8-H); ^13^C NMR (150 MHz; CD_3_OD) δ 9.7, 23.5, 25.7, 26.4, 30.4, 30.5, 30.57, 30.64, 33.1, 35.2, 41.0, 56.7, 113.8, 116.2, 123.6, 124.3, 145.9, 146.7; *m/z* [HRMS CI+] found [MH]^+^ 292.22715. C_18_H_30_NO_2_ requires 292.22765.

**(1*RS*,3*S*,4*R*)-1-(1,3-Benzodioxol-5-ylmethyl)-3-methyl-1,2,3,4-tetrahydroisoquinoline-4,6-diol** (**25).** Compound **25** was prepared according to procedure **A** using metaraminol (+) bitartrate salt (50 mg, 0.16 mmol) and 1,3-benzodioxole-5-acetaldehyde [(Pesnot, et al., 2012)](#_ENREF_33) (40 mg, 0.24 mmol). The product was purified by preparative HPLC (gradient **1**, retention time 20.5 min) to give **25** as a diastereomeric mixture of a and b in a ratio of approximately 3:2 (46 mg, 92%). ^1^H NMR (600 MHz; CD_3_OD) δ 1.37 (2H, d, *J* = 6.6 Hz, CH_3b_), 1.50 (3H, d, *J* = 6.5 Hz, CH_3a_), 2.94–3.70 (3H_a_ and 2H_b_, m, 3-H_a_, 3-H_b_, NCHC*H*_2a_, NCHC*H*_2b_), 4.50 (0.7H_b_, d, *J* = 6.8 Hz, 4-H_b_), 4.58 (1H_a_, d, *J* = 9.8 Hz, 4-H_a_), 4.65 (0.7H_b_, t, *J* = 7.1 Hz, 1-H_b_), 4.76‒4.81 (1H_a_, m, 1-H_a_), 5.96 (1.4H_b_, s, OCH_2b_O), 5.97 (2H_a_, s, OCH_2a_O), 6.70‒7.08 (5H_a_ and 4H_b_, m, 5-H_a_, 5-H_b_, 7-H_a_, 7-H_b_, 8-H_b_, 2'-H_a_, 2'-H_b_, 5'-H_a_, 5'-H_b_, 6'-H_a_ and 6'-H_b_), 7.22 (1H_a_, d, *J* = 8.6 Hz, 8-H_a_); ^13^C NMR (150 MHz; CD_3_OD) δ 15.1, 16.1, 41.2, 41.3, 53.0, 56.4, 56.8, 58.8, 70.1, 70.2, 102.54, 102.55, 109.6, 109.7, 110.6, 110.7, 114.2, 115.9, 116.8, 116.9, 122.8, 123.3, 123.9, 124.1, 128.1, 129.1, 130.1, 130.2, 136.9, 139.7, 148.70, 148.72, 149.7, 149.8, 158.9, 159.0; *m/z* [ES+] 314 (MH^+^, 20%), 296 (MH^+^-H_2_O, 100), 160 (43); *m/z* [HRMS ES+] found [MH]^+^ 314.1391. C_18_H_20_NO_4_ requires 314.1392.

**(1*RS*,3*S*)-1-(1,3-Benzodioxol-5-ylmethyl)-6,7-dihydroxy-1,2,3,4-tetrahydroisoquinoline-3-carboxylic acid (26).** Compound **26** was prepared according to procedure **A** using L-DOPA (30 mg, 0.15 mmol) and 1,3-benzodioxole-5-acetaldehyde [(Pesnot, et al., 2012)](#_ENREF_33) (38 mg, 0.23 mmol). The product was purified by preparative HPLC (gradient **1**, retention time 18.0 min) to give **26** as a 1:1 diastereomeric mixture (50 mg, 96%). ^1^H NMR (600 MHz; CD_3_OD) δ 2.96 (1H_b_, dd, *J* = 14.6 and 8.6 Hz, NCHCH*H*_b_), 3.14 (1H_a_, dd, *J* = 15.0 and 10.4 Hz, 4-*H*H_a_), 3.10–3.23 (2H_a_ and 2H_b_, m, 4-H_2b_ and NCHC*H*_2a_), 3.26‒3.31 (1H_a_, m, 4-H*H*_a_), 3.54 (1H_b_, dd, *J* = 14.6 and 5.0 Hz, NCHC*H*H_b_), 4.05 (1H_b_, dd, *J* = 12.5 and 5.0 Hz, 3-H_b_), 4.34 (1H_a_, dd, *J* = 10.4 and 5.9 Hz, 3-H_a_), 4.64‒4.67 (1H_a_ and 1H_b_, m, 1-H_a_ and 1-H_b_), 5.95 (2H_b_, s, OCH_2b_O), 5.97 (2H_a_, s, OCH_2a_O), 6.19 (1H_a_, s, 8-H_a_), 6.62‒6.68 (4H, m), 6.79 (1H_b_, d, *J* = 7.9 Hz, 5'-H), 6.83–6.92 (4H, m); ^13^C NMR (150 MHz; CD_3_OD) δ 29.1, 29.8, 40.5, 41.1, 52.3, 56.4, 57.5, 58.7, 102.5, 102.6, 109.5, 109.7, 110.6, 110.7, 113.4, 114.7, 116.0, 116.1, 122.1, 122.8, 123.2, 123.4, 123.9, 124.1, 130.1, 130.3, 145.6, 146.3, 147.0, 147.1, 148.6, 148.8, 149.6, 149.9, 171.4, 171.5; *m/z* [ES+] 314 (MH^+^, 100%), 208 (65); *m/z* [HRMS ES+] found [MH]^+^ 344.1130. C_18_H_18_NO_6_ requires 344.1134.

**1-(1,3-Benzodioxol-5-ylmethyl)-1,2,3,4-tetrahydroisoquinoline-6-ol** (**27).** Compound **27** was prepared according to procedure **A** using 2-(3-hydroxyphenyl)ethylamine [(Pesnot, et al., 2012)](#_ENREF_33) (50 mg, 0.36 mmol) and 1,3-benzodioxole-5-acetaldehyde[(Pesnot, et al., 2012)](#_ENREF_33) (60 mg, 0.37 mmol). The product was purified by preparative HPLC (gradient **2,** retention time 13.0 min) to give **27** as a colourless oil (25 mg, 25%). ^1^H NMR (600 MHz; D_2_O) δ 2.92–3.07 (3H, m, 4-H_2_, NCHC*H*H), 3.23‒3.29 (1H, m, 3-*H*H), 3.35 (1H, dd, *J* = 14.6 and 5.9 Hz, NCHCH*H*), 3.46 (1H, app. quintet, *J* = 6.4 Hz, 3-H*H*), 4.63 (1H, dd, *J* = 8.8 and 5.9 Hz, 1-H), 5.89 (2H, s, OCH_2_O), 6.67‒6.73 (3H, m, 5-H, 7-H, 6'-H), 6.75 (1H, d, *J* = 1.3 Hz, 2'-H), 6.81 (1H, d, *J* = 7.9 Hz, 5'-H), 7.02 (1H, d, *J* = 8.5 Hz, 8-H); ^13^C NMR (150 MHz; D_2_O) δ 27.0, 41.2, 41.3, 58.5, 103.4, 111.0, 111.7, 116.5, 117.1, 125.1, 125.2, 130.4, 130.8, 135.3, 148.9, 149.9, 157.6; *m/z* [ES+] 284 (MH^+^, 60%), 267 (62), 237 (20), 148 (100); *m/z* [HRMS ES+] found [MH]^+^ 284.1288. C_17_H_18_NO_3_ requires 284.1287.

**3-(2-Aminoethyl)-4-bromophenol**. The reaction was carried out under anhydrous conditions. To a solution of 2-(2-bromo-5-methoxyphenyl)ethylamine [(Liang, et al., 2010)](#_ENREF_34) (120 mg, 0.52 mmol) in dichloromethane (20 mL) at –78 °C, boron tribromide (1.3 mL, 1.3 mmol; 1 M solution in hexane) was added. The mixture was warmed to rt and stirred for 20 h, then cooled to –78 °C and water (50 mL) added dropwise. The aqueous layer was washed with dichloromethane (3 × 50 mL), filtered and concentrated under reduced pressure to give 3-(2-aminoethyl)-4-bromophenol[(Chao, et al., 2005)](#_ENREF_47) as a colourless oil (110 mg, 97%). ^1^H NMR (600 MHz; D_2_O) δ 3.09 (2H, t, *J* = 7.5 Hz, C*H*_2_CH_2_NH_2_), 3.29 (2H, t, *J* = 7.5 Hz, CH_2_C*H*_2_NH_2_), 6.77 (1H, dd, *J* = 8.7 and 3.0 Hz, 6-H), 6.90 (1H, d, *J* = 3.0 Hz, 2-H), 7.52 (1H, d, *J* = 8.7 Hz, 5-H); ^13^C NMR (150 MHz; D_2_O) δ 33.9, 39.7, 114.4, 117.1, 118.7, 134.6, 137.7, 156.1; *m/z* [HRMS ES+] found [MH]^+^ 216.0030. C_8_H_11_^79^BrNO requires 216.0024.

**1-(1,3-Benzodioxol-5-ylmethyl)-5-bromo-1,2,3,4-tetrahydroisoquinoline-8-ol (28).** Compound **28** was prepared according to procedure **A** using 3-(2-aminoethyl)-4-bromophenol (25 mg, 115 μmol) and 1,3-benzodioxole-5-acetaldehyde [(Pesnot, et al., 2012)](#_ENREF_33) (15 mg, 91 μmol). The product was purified by preparative HPLC (gradient **2**, retention time 14.2 min) to give **28** as a colourless oil (5.5 mg, 17%). ^1^H NMR (600 MHz; CD_3_OD) δ 2.92–3.03 (2H, m, 4-*H*H, NCHC*H*H), 3.08–3.11 (1H, m, 4-H*H*), 3.37‒3.42 (1H, m, 3-*H*H), 3.48 (1H, dd, *J* = 15.2 and 3.4 Hz, NCHCH*H*), 3.56‒3.64 (1H, m, 3-H*H*), 4.86‒4.88 (1H, m, 1-H), 5.97 (2H, s, OCH_2_O), 6.76 (1H, d, *J* = 8.6 Hz, 7-H), 6.82‒6.85 (2H, m, 5'-H and 6'-H), 6.87 (1H, s, 2'-H), 7.44 (1H, d, *J* = 8.6 Hz, 6-H); ^13^C NMR (150 MHz; CD_3_OD) δ 27.5, 37.3, 37.5, 54.3, 102.6, 109.8, 110.2, 114.6, 116.1, 122.7, 123.6, 130.4, 132.8, 133.8, 148.8, 149.9, 154.9; *m/z* [ES+] 364 (M[^81^Br]H^+^, 38%), 362 (M[^79^Br]H^+^, 40), 229 (100), 226 (82); *m/z* [HRMS ES+] found [MH]^+^ 362.0371. C_17_H_17_^79^BrNO_3_ requires 362.0392.

**1-(1,3-Benzodioxol-4-yl)-6,7-dihydroxy-1,2,3,4-tetrahydroisoquinoline (29).** Compound **29** was prepared according to procedure **A** using dopamine hydrochloride (30 mg, 0.16 mmol) and 2,3-(methylenedioxy)benzaldehyde (36 mg, 0.24 mmol). The product was purified by preparative HPLC (gradient **1**, retention time 15.6 min) to give **29** as a colourless oil (45 mg, 99%). ^1^H NMR (600 MHz; DMSO-d_6_) δ 2.86 (1H, ddd, *J* = 17.2, 5.7 and 5.7 Hz, 4-*H*H), 2.96–3.02 (1H, m, 4-H*H*), 3.29‒3.40 (2H, m, 3-H_2_), 5.54 (1H, s, 1-H), 5.88 (1H, d, *J* = 0.7 Hz, OC*H*HO), 5.91 (1H, d, *J* = 0.7 Hz, OCH*H*O), 6.14 (1H, s, 8-H)*,* 6.52‒6.56 (2H, m, 5-H and 6'-H)*,* 6.76 (1H, t, *J* = 7.7 Hz, 5'-H), 6.80 (1H, dd, *J* = 7.7 and 1.3 Hz, 4'-H); ^13^C NMR (150 MHz; DMSO-d_6_) δ 23.0, 39.1, 52.6, 100.5, 108.2, 112.2, 113.4, 116.5, 119.6, 120.7, 120.9, 121.7, 143.4, 144.5, 145.1, 146.9; *m/z* [ES+] 286 (MH^+^, 100%), 269 (46), 239 (23); *m/z* [HRMS ES+] found [MH]^+^ 286.1087. C_16_H_16_NO_4_ requires 286.1079.

**(1RS,4*R*)-1-(1,3-Benzodioxol-4-yl)-1,2,3,4-tetrahydroisoquinoline-4,6,7-triol (30).** Compound **30** was prepared according to procedure **A** using (-)-norepinephrine (30 mg, 0.18 mmol) and 2,3-(methylenedioxy)benzaldehyde (40 mg, 0.27 mmol). The product was purified by preparative HPLC (gradient **1**, retention time 16.8 min) to give **30** as a diastereomeric mixture in a ratio of approximately 3:2 (45 mg, 83%). ^1^H NMR (600 MHz; CD_3_OD) δ 3.27–3.30 (0.7H_b_, m, 3-*H*H_b_), 3.46‒3.52 (1H_a_ and 0.7H_b_, m, 3-*H*H_a_ and 3-H*H*_b_), 3.57 (1H_a_, dd, *J* = 12.7 and 2.9 Hz, 3-H*H*_a_), 4.83-4.90 (1H_a_ and 0.7H_b_, m, 4-H_a_ and 4-H_b_), 5.64 (1H_a_, app. s, 1-H_a_)*,* 5.73 (0.7H_b_, app. s, 1-H_b_)*,* 6.02 (1H_a_, d, *J* = 0.9 Hz, OC*H*H_a_O), 6.04 (0.7H_b_, d, *J* = 0.9 Hz, OC*H*H_b_O), 6.05 (1H_a_, d, *J* = 0.9 Hz, OCH*H*_a_O), 6.08 (0.7H_b_, d, *J* = 0.9 Hz, OCH*H*_b_O), 6.26 (1H_a_, s, 8-H_a_)*,* 6.34 (0.7H_b_, s, 8-H_b_)*,* 6.50 (0.7H_b_, d, *J* = 7.9 Hz, 6'-H_b_)*,* 6.80 (1H, dd, *J* = 7.0 and 2.1 Hz, 6'-H_a_), 6.86‒6.97 (3H_a_ and 2.1H_b_, m, 5-H_a_, 5-H_b_, 4'-H_a_, 4'-H_b_, 5'-H_a_ and 5'-H_b_); ^13^C NMR (150 MHz; CD_3_OD) δ 46.7, 49.7, 54.1, 55.9, 63.5, 63.6, 103.17, 103.22, 110.9, 111.0, 114.3, 114.5, 116.3, 116.6, 118.6, 118.8, 121.7, 122.7, 123.3, 123.46, 123.47, 123.6, 127.3, 127.7, 147.2, 147.4, 147.7, 147.8, 147.9, 149.5 (signals superimposed); *m/z* [ES+] 284 ([M-OH]^+^, 100%), 162 (18); *m/z* [HRMS ES+] found [M-OH]^+^ 284.0929. C_16_H_14_NO_4_ requires 284.0923.

**(1*R*,4*R*)-1-(1,3-Benzodioxol-4-yl)-2-methyl-1,2,3,4-tetrahydroisoquinoline-4,6,7-triol (31)** and **(1*S*,4*R*)-1-(1,3-Benzodioxol-4-yl)-2-methyl-1,2,3,4-tetrahydroisoquinoline-4,6,7-triol (32).** Compounds **31** and **32** were prepared according to procedure **A** using (-)-epinephrine (50 mg, 0.27 mmol) and 2,3-(methylenedioxy)benzaldehyde (61 mg, 0.41 mmol). The product was purified by preparative HPLC (gradient **1**) to give **31** (49 mg, 57%, retention time 15.2 min) and **32** (33 mg, 38%, retention time 15.9 min) as colourless oils. Major isomer **31**: [α]^25^_D_ +25.4 (c 1.0 in MeOH); ^1^H NMR (600 MHz; CD_3_OD) δ 2.96 (3H, s, CH_3_), 3.75 (2H, br d, *J* = 1.6 Hz, 3-H_2_), 5.01 (1H, br s, 4-H), 5.57 (1H, s, 1-H)*,* 6.00 (1H, app. s, OC*H*HO), 6.01 (1H, app. s, OCH*H*O), 6.30 (1H, s, 8-H)*,* 6.82 (1H, d, *J* = 7.9 Hz, 6'-H), 6.97 (1H, s, 5-H), 6.99 (1H, t, *J* = 7.9 Hz, 5'-H), 7.05 (1H, d, *J* = 7.9 Hz, 4'-H); ^13^C NMR (150 MHz; CD_3_OD) δ 42.9, 59.2, 63.5, 65.2, 102.5, 111.1, 114.9, 116.0, 116.6, 123.3, 123.5, 123.9, 125.5, 145.2, 146.0, 147.5, 148.4; *m/z* [HRMS ES+] found [MNa]^+^ 338.1009. C_17_H_17_NO_5_Na requires 338.1004. Minor isomer **32**: [α]^25^_D_ -52.8 (c 1.0 in MeOH); ^1^H NMR (600 MHz; CD_3_CN) δ 2.82 (3H, s, CH_3_), 3.33 (1H, br d, *J* = 9.5 Hz, 3-H*H*), 3.61 (1H, br m, 3-*H*H), 4.88 (1H, br s, 4-H), 5.50 (1H, s, 1-H)*,* 6.03 (2H, s, OCH_2_O), 6.28‒6.50 (2H, m, 5-H and 6'-H), 6.85 (1H, t, *J* = 7.9 Hz, 5'-H), 6.94 (1H, d, *J* = 7.9 Hz, 4'-H), 7.00 (1H, s, 5-H); ^13^C NMR (150 MHz; CD_3_CN) δ 40.4, 54.6, 59.1, 62.9, 101.5, 109.4, 112.8, 115.5, 117.5 (some signals not observed under solvent peak), 121.9, 145.2, 145.3, 147.2, 147.6; *m/z* [ES+] 316 (MH^+^, 100%), 299 (48), 298 (100), 227 (33), 199 (48), 176 (42); *m/z* [HRMS ES+] found [MH]^+^ 316.1177. C_17_H_18_NO_5_ requires 316.1185.

**1-(1,3-Benzodioxol-4-yl)-1,2,3,4-tetrahydroisoquinoline-6,7,8-triol (33).** Compound **33** was prepared according to procedure **A** using 5-hydroxydopamine hydrochloride (30 mg, 0.15 mmol) and 2,3-(methylenedioxy)benzaldehyde (32 mg, 0.21 mmol). To limit excessive oxidation of the amine, the reaction was carried out at room temperature and under argon. The product was purified by preparative HPLC (gradient **1**, retention time 15.0 min) to give **33** as a pale yellow oil (12.5 mg, 28%). ^1^H NMR (600 MHz; CD_3_OD) δ 2.92‒2.97 (1H, m, 4-*H*H), 3.04–3.10 (1H, m, 4-H*H*), 3.22‒3.29 (1H, m, 3-*H*H), 3.35–3.43 (1H, m, 3-H*H*), 5.84 (1H, s, 1-H)*,* 6.04 (1H, d, *J* = 1.1 Hz, OC*H*HO), 6.07 (1H, d, *J* = 1.1 Hz, OCH*H*O), 6.31 (1H, s, 5-H), 6.41 (1H, d, *J* = 7.8 Hz, 6'-H), 6.87 (1H, t, *J* = 7.8 Hz, 5'-H), 6.87 (1H, d, *J* = 7.8 Hz, 4'-H); ^13^C NMR (150 MHz; CD_3_OD) δ 25.5, 38.6, 50.4, 103.1, 107.1, 109.3, 110.4, 119.1, 122.8, 123.4, 123.9, 133.1, 144.6, 147.4, 148.1, 149.3; *m/z* [ES+] 301 (M^+^, 37%), 180 (100); *m/z* [HRMS EI+] found M^+^ 301.09410. C_16_H_15_NO_5_ requires 301.09447.

**1-(1,3-Benzodioxol-4-yl)-6-hydroxy-1,2,3,4-tetrahydroisoquinoline (34).** Compound **34** was prepared according to procedure **A** using 2-(3-hydroxyphenyl)ethylamine [(Pesnot, et al., 2012)](#_ENREF_33) (65 mg, 0.47 mmol) and 2,3-(methylenedioxy)benzaldehyde (52 mg, 0.35 mmol). The product was purified by preparative HPLC (gradient **2**, retention time 12.9 min) to give **34** as a colourless oil (74 mg, 79%). ^1^H NMR (600 MHz; D_2_O) δ 3.09–3.24 (2H, m, 4-H_2_), 3.46‒3.60 (2H, m, 3-H_2_), 5.79 (1H, s, 1-H), 5.96 (1H, d, *J* = 0.8 Hz OC*H*HO), 5.99 (1H, d, *J* = 0.8 Hz, OCH*H*O), 6.68 (1H, d, *J* = 7.9 Hz, 6'-H)*,* 6.72 (1H, dd, *J* = 8.5 and 2.6 Hz, 7-H)*,* 6.80‒6.84 (2H, m, 5-H, 8-H), 6.91 (1H, t, *J* = 7.9 Hz, 5'-H), 6.99 (1H, d, *J* = 7.9 Hz, 4'-H); ^13^C MR (150 MHz; D_2_O) δ 25.3, 40.5, 54.8, 102.3, 110.7, 115.3, 115.4, 117.9, 122.4, 123.0, 123.3, 129.7, 134.3, 146.5, 148.3, 156.1; *m/z* [ES+] 270 (MH^+^, 100%), 253 (22), 223 (18); *m/z* [HRMS ES+] found [MH]^+^ 270.1122. C_16_H_16_NO_3_ requires 270.1130.

**1-(1,3-Benzodioxol-4-yl)-5-bromo-1,2,3,4-tetrahydroisoquinoline-8-ol (35).** Compound **35** was prepared according to procedure **A** using 3-(2-aminoethyl)-4-bromophenol (25 mg, 116 μmol) and 2,3-(methylenedioxy)benzaldehyde (14 mg, 93 μmol). The product was purified by preparative HPLC (gradient **1**, retention time 16.6 min) to give **35** as a colourless oil (2.5 mg, 8%). ^1^H NMR (600 MHz; CD_3_OD) δ 3.02–3.08 (1H, m, 4-*H*H), 3.17–3.22 (1H, m, 4-H*H*), 3.32–3.37 (1H, m, 3-*H*H), 3.48 (1H, dd, *J* = 13.1 and 5.7 Hz, 3-H*H*), 5.89 (1H, s, 1-H), 6.04 (1H, d, *J* = 1.0 Hz, OC*H*HO), 6.09 (1H, d, *J* = 1.0 Hz, OCH*H*O), 6.44 (1H, d, *J* = 7.9 Hz, 6'-H), 6.68 (1H, d, *J* = 8.7 Hz, 7-H), 6.83 (1H, t, *J* = 7.9 Hz, 5'-H), 6.91 (1H, d, *J* = 7.9 Hz, 4'-H), 7.49 (1H, d, *J* = 8.7 Hz, 6-H); ^13^C NMR (150 MHz; CD_3_OD) δ 27.5, 38.0, 50.5, 103.2, 110.7, 114.2, 115.9, 117.1, 119.0, 123.1, 123.3, 133.7, 134.2, 147.6, 149.4, 155.2; *m/z* [ES+] 350 (M[^81^Br]H^+^, 98%), (M[^79^Br]H^+^, 100), 332 (20), 330 (18), 252 (32); *m/z* [HRMS ES+] found [MH]^+^ 348.0230. C_16_H_15_^79^BrNO_3_ requires 348.0235.

**2-(2-Chloro-5-methoxyphenyl)acetonitrile.** To a solution of 3-methoxyphenylacetonitrile (2.00 g, 13.6 mmol) in acetonitrile (50 mL) was added *N*-chlorosuccinimide (2.00 g, 15.0 mmol) by portion-wise addition and 300 μL of trifluoroacetic acid (4 mmol). The reaction was stirred at 40 ºC for 5 h, concentrated under reduced pressure and purified using flash silica chromatography (10% EtOAc in hexane) to give the titled compound [(Pierre and Baudoin, 2011)](#_ENREF_48) (2.09 g, 85%). *ν*_max_ (neat)/cm^–1^ 2944, 2252, 1606, 1579, 1477; ^1^H NMR (600 MHz; CDCl_3_) δ 3.80 (2H, s, CH_2_), 3.82 (3H, s, OCH_3_), 6.83 (1H, dd, *J* = 8.8 and 2.9 Hz, 4-H), 7.05 (1H, d, *J* = 2.9 Hz, 6-H), 7.30 (1H, d, *J* = 8.8 Hz, 3-H); ^13^C NMR (150 MHz; CDCl_3_) δ 22.4, 55.8, 115.2, 115.3, 117.0, 124.7, 129.0, 130.6, 158.8; *m/z* [HRMS EI] found M^+^ 181.02916. C_9_H_8_^35^ClNO requires 181.02889.

**2-(2-Iodo-5-methoxyphenyl)acetonitrile.** To a solution of 3-methoxyphenylacetonitrile (2.00 g, 13.6 mmol) in acetonitrile (50 mL) was added *N*-iodosuccinimide (3.20 g, 14.0 mmol) by portion-wise addition and 300 µL of trifluoroacetic acid (4 mmol). The reaction was stirred at rt for 40 h, concentrated under reduced pressure and purified using flash silica chromatography (5% EtOAc in hexane) to give the titled compound (3.00 g, 81%). *ν*_max_ (neat)/cm^–1^ 2919, 2241, 1585, 1470, 1454; ^1^H NMR (600 MHz; CDCl_3_) δ 3.78 (2H, s, CH_2_), 3.82 (3H, s, OCH_3_), 6.64 (1H, dd, *J* = 8.7 and 2.9 Hz, 4-H), 7.10 (1H, d, *J* = 2.9 Hz, 6-H), 7.72 (1H, d, *J* = 8.7 Hz, 3-H); ^13^C NMR (150 MHz; CDCl_3_) δ 30.1, 55.7, 87.2, 115.3, 116.0, 117.3, 134.2, 140.4, 160.5; *m/z* [HRMS EI] found M^+^ 272.96474. C_9_H_8_INO requires 272.96451.

**2-(2-Chloro-5-methoxyphenyl)ethylamine.** The reaction was carried out under anhydrous conditions. To a solution of 2-(2-chloro-5-methoxyphenyl)acetonitrile (1.00 g, 5.50 mmol) in THF (30 mL) was added BF_3_.Et_2_O (200 μL, 1.60 mmol) followed by the dropwise addition of B_2_H_6_.SMe_2_ (2 M solution; 6.90 mL, 13.8 mmol). The reaction was heated at reflux for 3 h, cooled to 0 ºC, then 20% HCl solution (70 mL) was added over 1 h, followed by stirring at rt for 2 h. The mixture was basified to a pH of 13 and the aqueous layer was extracted with CH_2_Cl_2_ (3 × 100 mL). The organic layers were combined and concentrated under reduced pressure to give the titled compound[(Mohr, et al., 2006)](#_ENREF_49) (440 mg, 43%) as an oil. ^1^H NMR (600 MHz; CD_3_OD) δ 2.84‒2.88 (4H, m, C*H*_2_C*H*_2_NH_2_), 3.77 (3H, s, OCH_3_), 6.77 (1H, dd, *J* = 8.8 and 3.0 Hz, 4-H), 6.86 (1H, d, *J* = 3.0 Hz, 6-H), 7.26 (1H, d, *J* = 8.8 Hz, 3-H); ^13^C NMR (150 MHz; CD_3_OD) δ 36.3, 40.8, 54.3, 112.9, 116.0, 124.6, 129.6, 137.6, 158.5; *m/z* [HRMS EI] found [MH]^+^ 186.06899. C_9_H_13_^35^ClNO requires 186.06857.

**2-(2-Iodo-5-methoxyphenyl)ethylamine.** The reaction was carried out under anhydrous conditions. To a solution of 2-(2-iodo-5-methoxyphenyl)acetonitrile (1.00 g, 3.66 mmol) in THF (20 mL) was added BF_3_.Et_2_O (200 µL, 1.60 mmol) followed by the dropwise addition of 2 M B_2_H_6_.SMe_2_ (4.50 mL, 9.00 mmol). The reaction was subsequently heated at reflux for 3 h, cooled to 0 ºC, and 20% HCl (70 mL) added to the reaction over 1 h. The mixture was stirred for 2 h at rt, was basified to pH 13, and the aqueous layer extracted with CH_2_Cl_2_ (3 × 100 mL). The organic layers were combined and concentrated under reduced pressure to give the titled compound (550 mg, 54%) as an oil. *ν*_max_ (neat)/cm^–1^ 3365, 3280, 2933, 2835, 1589, 1566, 1465; ^1^H NMR (600 MHz; CD_3_OD) δ 2.81‒2.86 (4H, m, C*H*_2_C*H*_2_NH_2_), 3.68 (3H, s, OCH_3_), 6.58 (1H, dd, *J* = 8.7 and 3.0 Hz, 4-H), 6.85 (1H, d, *J* = 3.0 Hz, 6-H), 7.68 (1H, d, *J* = 8.7 Hz, 3-H); ^13^C NMR (150 MHz; CD_3_OD) δ 42.9, 44.9, 55.8, 89.1, 115.3, 117.0, 141.2, 144.5, 161.7.

**2-(Benzo[d][1,3]dioxol-5-yl)-*N*-(2-chloro-5-methoxyphenethyl)acetamide).** To 2-(2-chloro-5-methoxyphenyl)ethylamine (200 mg, 1.08 mmol) in CH_2_Cl_2_ was added 3,4-(methylenedioxy)phenylacetic acid (207 mg, 1.30 mmol), *N*,*N*-dicyclohexylcarbodiimide (DCC) (267 mg, 1.30 mmol) and dimethylaminopyridine (DMAP) (16 mg, 0.13 mmol). The reaction was stirred for 3 h at rt, concentrated under reduced pressure and the crude product purified by flash silica chromatography (40% EtOAc in hexane) to yield the titled compound (375 mg, 99%) as an oil. ^1^H NMR (600 MHz; CDCl_3_) δ 2.86 (2H, t, *J* = 6.7 Hz, C*H*_2_CH_2_N), 3.45 (2H, s, COC*H*_2_), 3.49 (2H, app. q, *J* = 6.7 Hz, CH_2_C*H*_2_N), 3.76 (3H, s, OCH_3_), 5.43 (1H, br s, NH), 5.96 (2H, s, OCH_2_O), 6.62 (1H, d, *J* = 7.9 Hz, 6'-H), 6.66 (2H, m, 6-H and 2'-H), 6.70 (1H, dd, *J* = 8.8 and 3.0 Hz, 4-H), 6.74 (1H, d, *J* = 7.9 Hz, 5'-H)*,* 7.21 (1H, d, *J* = 8.8 Hz, 3-H); ^13^C NMR (150 MHz; CDCl_3_) δ 33.4 39.3, 43.6, 55.6, 101.3, 108.8, 109.9, 113.5, 116.5, 122.8, 125.4, 128.2, 130.3, 137.4, 147.0, 148.2, 158.4, 171.3; *m/z* [ES+] 370 (MNa^+^, 36%), 350 (M[^37^Cl]H^+^, 33), 348 (M[^35^Cl]H^+^, 100), 169 (35); *m/z* [HRMS ES+] found [MH]^+^ 348.1010. C_18_H_19_^35^ClNO_4_ requires 348.1003.

**(2-(Benzo[d][1,3]dioxol-5-yl)-*N*-(2-bromo-5-methoxyphenethyl)acetamide).** To 2-(2-bromo-5-methoxyphenyl)ethylamine[(Liang, et al., 2010)](#_ENREF_34) (110 mg, 0.48 mmol) in CH_2_Cl_2_ was added 3,4-(methylenedioxy)phenylacetic acid (80 mg, 0.50 mmol), DCC (103 mg, 0.50 mmol) and DMAP (5 mg, 0.04 mmol). The reaction was stirred for 3 h at rt, concentrated under reduced pressure and the crude product purified by flash silica chromatography (40% EtOAc in hexane) to yield the titled compound (180 mg, 96%) as an oil. ^1^H NMR (600 MHz; CDCl_3_) δ 2.86 (2H, t, *J* = 6.8 Hz, C*H*_2_CH_2_N), 3.44 (2H, s, COC*H*_2_), 3.48 (2H, app. q, *J* = 6.8 Hz, CH_2_C*H*_2_N), 3.75 (3H, s, OCH_3_), 5.50 (1H, br.s, NH), 5.94 (2H, s, OCH_2_O), 6.61‒6.67 (4H, m, 4-H, 6-H, 2'-H and 6'-H), 6.73 (1H, d, *J* = 7.8 Hz, 5'-H)*,* 7.37 (1H, d, *J* = 8.6 Hz, 3-H); ^13^C NMR (150 MHz; CDCl_3_) δ 35.8, 39.4, 43.6, 55.5, 101.2, 108.7, 109.9, 113.9, 114.9, 116.7, 123.0, 128.3, 133.5, 139.1, 147.0, 148.2, 159.1, 171.3; *m/z* [ES+] 416 (M[^81^Br]Na^+^, 32%), 414 (M[^79^Br]Na^+^, 35), 394 (M[^81^Br]H^+^, 100), 392 (M[^79^Br]H^+^, 96), 232 (70), 230 (72), 135 (35); *m/z* [HRMS CI+] found [MH]^+^ 392.04911. C_18_H_19_^79^BrNO_4_ requires 392.04975.

**(2-(Benzo[d][1,3]dioxol-5-yl)-*N*-(2-iodo-5-methoxyphenethyl)acetamide).** To 2-(2-iodo-5-methoxyphenyl)ethylamine (90 mg, 0.32 mmol) in CH_2_Cl_2_ was added 3,4-(methylenedioxy)phenylacetic acid (62 mg, 0.39 mmol), DCC (80 mg, 0.39 mmol) and DMAP (5 mg, 0.04 mmol). The reaction was stirred for 3 h at rt, concentrated under reduced pressure and the crude product purified by flash silica chromatography (40% EtOAc in hexane) to yield the titled compound (100 mg, 74%) as an oil. ^1^H NMR (600 MHz; CDCl_3_) δ 2.86 (2H, t, *J* = 6.9 Hz, C*H*_2_CH_2_N), 3.42‒3.48 (4H, m, COC*H*_2_ and CH_2_C*H*_2_N), 3.74 (3H, s, OCH_3_), 5.51 (1H, br s, NH), 5.92 (2H, s, OCH_2_O), 6.48‒6.70 (4H, m, 4-H, 6-H, 2'-H and 6'-H), 6.72 (1H, d, *J* = 7.9 Hz, 5'-H)*,* 7.63 (1H, d, *J* = 8.7 Hz, 3-H); ^13^C NMR (150 MHz; CDCl_3_) δ 39.6, 40.4, 43.7, 55.5, 89.0, 101.2, 108.4, 109.9, 114.6, 116.6, 122.7, 128.2, 140.3, 142.5, 147.0, 148.2, 160.1, 171.4; *m/z* [HRMS ES+] found [MNa]^+^ 462.0179. C_18_H_18_INNaO_4_ requires 462.0178.

**1-(1,3-Benzodioxol-5-ylmethyl)-5-chloro-8-methoxy-1,2,3,4-tetrahydroisoquinoline 36.** Compound **36** was prepared from 2-(benzo[d][1,3]dioxol-5-yl)-*N*-(2-chloro-5-methoxyphenethyl)acetamide) (265 mg, 0.757 mmol) according to procedure **B**. The product was purified by preparative HPLC (gradient **2**, retention time 15.0 min) to give **36** as a colourless oil (49 mg, 19%). ^1^H NMR (600 MHz; CD_3_OD) δ 2.99 (1H, dd, *J* = 15.1 and 10.1 Hz, NCHC*H*H), 3.03–3.08 (1H, m, 4-H*H*), 3.15 (1H, ddd, *J* = 18.2, 5.7 and 2.3 Hz, 4-*H*H), 3.36 (1H, dd, *J* = 15.1 and 4.5 Hz, NCHCH*H*), 3.44 (1H, ddd, *J* = 13.0, 7.1 and 2.3 Hz, 3-*H*H), 3.62‒3.68 (1H, m, 3-H*H*), 3.89 (3H, s, CH_3_), 4.88‒4.92 (1H, m, 1-H), 5.97 (2H, m, OCH_2_O), 6.80 (1H, dd, *J* = 7.9 and 1.3 Hz, 6'-H), 6.79‒6.86 (2H, m, 2'-H, 5'-H)*,* 7.00 (1H, d, *J* = 8.8 Hz, 7-H), 7.45 (1H, d, *J* = 8.8 Hz, 6-H); ^13^C NMR (150 MHz; CD_3_OD) δ 24.5, 37.0, 37.7, 53.8, 56.5, 102.6, 109.7, 110.3, 111.5, 123.6, 126.6, 130.3, 130.7, 131.0, 131.3, 148.8, 149.9, 156.0; *m/z* [ES+] 332 (M[^35^Cl]H^+^, 8%), 210 (18), 198 (32), 196 (100), 181 (30); *m/z* [HRMS ES+] found [MH]^+^ 332.1064. C_18_H_19_^35^ClNO_3_ requires 332.1053.

**1-(1,3-Benzodioxol-5-ylmethyl)-5-bromo-8-methoxy-1,2,3,4-tetrahydroisoquinoline 37.** Compound **37** was prepared from 2-(benzo[d][1,3]dioxol-5-yl)-*N*-(2-bromo-5-methoxyphenethyl)acetamide) (120 mg, 0.305 mmol) according to procedure **B**. The product was purified by preparative HPLC (gradient **2**, retention time 15.5 min) to give **37** as a colourless oil (21 mg, 19%). ^1^H NMR (600 MHz; CD_3_OD) δ 2.97–3.04 (2H, m, 4-*H*H, NCHC*H*H), 3.11–3.16 (1H, m, 4-H*H*), 3.34 (1H, dd, *J* = 15.1 and 4.1 Hz, NCHCH*H*), 3.44 (1H, ddd, *J* = 13.0, 7.1 and 2.3 Hz, 3-*H*H), 3.62‒3.68 (1H, m, 3-H*H*), 3.90 (3H, s, CH_3_), 4.89‒4.92 (1H, m, 1-H), 5.97 (2H, s, OCH_2_O), 6.80 (1H, dd, *J* = 7.9 and 1.3 Hz, 6'-H), 6.83‒6.86 (2H, m, 2'-H, 5'-H), 6.95 (1H, d, *J* = 8.8 Hz, 7-H), 7.63 (1H, d, *J* = 8.8 Hz, 6-H); ^13^C NMR (150 MHz; CD_3_OD) δ 26.1, 37.2, 37.8, 53.8, 56.5, 102.6 109.7, 110.3, 112.0, 116.3, 123.6, 124.0, 130.3, 132.8, 134.1, 148.8, 149.9, 156.7; *m/z* [ES+] 380 (M[^81^Br]H^+^, 28%), 378 (M[^79^Br]H^+^, 27), 242 (100), 240 (98); *m/z* [HRMS ES+] found [MH]^+^ 376.0530. C_18_H_19_^79^BrNO_3_ requires 376.0548.

**1-(1,3-Benzodioxol-5-ylmethyl)-5-iodo-8-methoxy-1,2,3,4-tetrahydroisoquinoline 38.** Compound **38** was prepared from 2-(benzo[d][1,3]dioxol-5-yl)-*N*-(2-iodo-5-methoxyphenethyl)acetamide) (100 mg, 0.216 mmol) according to procedure **B**. The product was purified by preparative HPLC (gradient **2**) to give **38** as a colourless oil (2.0 mg, 2%). ^1^H NMR (600 MHz; CD_3_OD) δ 2.94–3.02 (2H, m, 4-*H*H, NCHC*H*H), 3.03–3.10 (1H, m, 4-H*H*), 3.42‒3.45 (1H, m, NCHCH*H*), 3.47‒3.53 (1H, m, 3-*H*H), 3.71‒3.77 (1H, m, 3-H*H*), 3.96 (3H, s, OCH_3_), 4.94‒4.98 (1H, m, 1-H), 6.04‒6.07 (2H, s, OCH_2_O), 6.86‒6.96 (4H, m, 7-H, 2'-H, 5'-H, 6'-H), 7.96 (1H, d, *J* = 8.7 Hz, 6-H); ^13^C NMR (125 MHz; CD_3_OD) δ 31.2, 36.19, 36.21, 52.2, 54.9, 89.1, 101.0, 108.2, 108.7, 111.0, 122.1, 122.4, 128.7, 134.1, 139.3, 147.2, 149.0, 156.0; *m/z* [ES+] 424 (MH^+^, 12%), 288 (100); *m/z* [HRMS ES+] found [MH]^+^ 424.0423. C_18_H_19_INO_3_ requires 424.0410.

**1-(1,3-Benzodioxol-4-bromo-5-ylmethyl)-5-chloro-8-methoxy-1,2,3,4-tetrahydroisoquinoline 39.** To a solution of **36** (6 mg, 54 μmol) in MeCN (10 mL) was added *N*-bromosuccinimide (11 mg, 60 μmol) and 1 drop of TFA. The mixture was stirred at rt for 3 h, concentrated to dryness and purified by preparative HPLC (gradient **2**, retention time 16.4 min) to give **39** as a colourless oil (2.6 mg, 35%). ^1^H NMR (600 MHz; CD_3_OD) δ 3.02‒3.09 (1H, m, 4-H*H*), 3.22‒3.27 (1H, m, NCHC*H*H), 3.30‒3.35 (1H, m, 4-*H*H), 3.41‒3.46 (1H, m, NCHCH*H*), 3.57‒3.64 (1H, m, 3-H*H*), 3.73 (3H, s, OCH_3_), 3.77‒3.84 (1H, m, 3-H*H*), 5.10 (1H, dd, *J* = 8.9 and 6.1 Hz, 1-H), 6.017 (1H, app. s, OC*H*HO), 6.021 (1H, app. s, OCH*H*O), 6.82 (1H, s, 2'-H), 6.94 (1H, d, *J* = 8.9 Hz, 7-H), 7.14 (1H, s, 5'-H), 7.44 (1H, d, *J* = 8.9 Hz, 6-H); ^13^C NMR (150 MHz; CD_3_OD) δ 24.4, 37.1, 38.2, 51.6, 56.2, 103.6, 111.9, 114.0, 116.3, 118.1, 122.5, 126.6, 128.7, 131.0, 131.3, 149.4, 149.8, 155.3; *m/z* [ES+] 412 (M[^81^Br^35^Cl]H^+^, 95%), 410 (M[^79^Br^35^Cl]H^+^, 65), 314 (58), 214 (34), 212 (35), 198 (32), 196 (100); *m/z* [HRMS ES+] found [MH]^+^ 410.0161. C_18_H_18_^79^Br^35^ClNO_3_ requires 410.0159.

**1-(1,3-Benzodioxol-5-ylmethyl)-8-methoxy-1,2,3,4-tetrahydroisoquinoline 40.** To a solution of **37** (4.5 mg, 12 μmol) in anhydrous THF (10 mL) was added LiAlH_4_ (4.6 mg, 120 μmol) at -78 ºC. The reaction mixture was stirred for 1 h at 0 ºC, then purified by preparative HPLC (gradient **2**) to give **40** as a colourless oil (3.2 mg, 89%). ^1^H NMR (600 MHz; CD_3_OD) δ 2.97 (1H, dd, *J* = 15.0 and 8.6 Hz, NCHC*H*H), 3.08‒3.13 (1H, m, 4-*H*H), 3.28‒3.35 (2H, m, 3-*H*H and 4-H*H*), 3.39 (1H, dd, *J* = 15.0 and 3.9 Hz, NCHCH*H*), 3.56‒3.64 (1H, m, 3-H*H*), 3.91 (3H, s, OCH_3_), 4.86‒4.91 (1H, m, 1-H), 5.96 (2H, s, OCH_2_O), 6.80 (1H, dd, *J* = 7.9 and 1.3 Hz, 6'-H), 6.82‒6.85 (2H, m, 2'-H, 5'-H), 6.88 (1H, d, *J* = 8.0 Hz, 5-H), 6.95 (1H, d, *J* = 8.0 Hz, 7-H), 7.96 (1H, t, *J* = 8.0 Hz, 6-H); ^13^C NMR (125 MHz; CD_3_OD) δ 25.8, 37.8, 38.1, 54.3, 56.2, 102.6, 109.7, 110.0, 110.3, 121.0, 122.3, 123.6, 130.5, 130.6, 133.5, 148.7, 149.8, 157.3; *m/z* [ES+] 298 (MH^+^, 10%), 281 (18), 162 (100); *m/z* [HRMS ES+] found [MH]^+^ 298.1450. C_18_H_20_NO_3_ requires 298.1443.

**References**

Brossi, A., Rice, K.C., Mak, C.-P., Reden, J., Jacobson, A.E., Nimitkitpaisan, Y., Skolnick, P., and Daly, J. (1980). Mammalian alkaloids. 8. Synthesis and biological effects of tetrahydropapaveroline related 1-benzyltetrahydroisoquinolines. Journal of Medicinal Chemistry 23, 648-652.

Chao, H.J.U., Tuerdi, H.U., Herpin, T.U., Roberge, J.Y.U., Liu, Y.U., Lawrence, R.M.U., Rehfuss, R.P.U., Clark, C.G.U., Qiao, J.X.U., Gungor, T.U., et al. (2005). Urea antagonists of P2Y1 receptor useful in the treatment of thrombotic conditions (B.-M.S. COMPANY, ed.).

Hahn, G., and Stiehl, K. (1936). Über β-[Oxy-phenyl]-äthylamine und ihre Umwandlungen, IV. Mitteil.: Synthese von Tetrahydro-isochinolin- carbonsäuren und die spontane Decarboxylierung von α-Keto-säuren unter physiologischen Bedingungen. Berichte der deutschen chemischen Gesellschaft (A and B Series) 69, 2627-2654.

Hartman, G.D., Phillips, B.T., and Halczenko, W. (1985). Iminium ion mediated cyclizations of 4-aryl-1,4-dihydropyridines. Bridging with acetals, carbonyls, and thiocarbonyls. The Journal of Organic Chemistry 50, 2423-2427.

Liang, J.T., Liu, J., Shireman, B.T., Tran, V., Deng, X., and Mani, N.S. (2010). A practical synthesis of regioisomeric 6- and 7-methoxytetrahydro-3-benzazepines. Organic Process Research & Development 14, 380-385.

Mohr, P., Decker, M., Enzensperger, C., and Lehmann, J. (2006). Dopamine/serotonin receptor ligands. 121:  SAR studies on hexahydro-dibenz[d,g]azecines lead to 4-chloro-7-methyl-5,6,7,8,9,14-hexahydrodibenz[d,g]azecin-3-ol, the first picomolar D5-selective dopamine-receptor antagonist. Journal of Medicinal Chemistry 49, 2110-2116.

Pesnot, T., Gershater, M.C., Ward, J.M., and Hailes, H.C. (2012). The catalytic potential of Coptis japonica NCS2 revealed – Development and utilisation of a fluorescamine-based assay. Advanced Synthesis & Catalysis 354, 2997-3008.

Pierre, C., and Baudoin, O. (2011). Synthesis of polycyclic molecules by double C(sp2)−H/C(sp3)−H arylations with a single palladium catalyst. Organic Letters 13, 1816-1819.

Ruff, B.M., Bräse, S., and O’Connor, S.E. (2012). Biocatalytic production of tetrahydroisoquinolines. Tetrahedron Letters 53, 1071-1074.

Schöpf, C., and Salzer, W. (1940). Zur Frage der Biogenese der 1-Benzyl-1,2,3,4-tetrahydro-isochinolin-alkaloide. Die Synthese des 1-(3′,4′-Methylendioxy-benzyl)-6,7-dioxy-1,2,3,4-tetrahydro-isochinolins unter zellmöglichen Bedingungen. Justus Liebigs Annalen der Chemie 544, 1-30.
